# Unaltered Fungal Burden and Lethality in Human CEACAM1-Transgenic Mice During *Candida albicans* Dissemination and Systemic Infection

**DOI:** 10.3389/fmicb.2019.02703

**Published:** 2019-11-26

**Authors:** Esther Klaile, Mario M. Müller, Cristina Zubiría-Barrera, Saskia Brehme, Tilman E. Klassert, Magdalena Stock, Adrian Durotin, Tien D. Nguyen, Sabina Feer, Bernhard B. Singer, Peter F. Zipfel, Sven Rudolphi, Ilse D. Jacobsen, Hortense Slevogt

**Affiliations:** ^1^Host Septomics Group, Centre for Innovation Competence (ZIK) Septomics, University Hospital Jena, Jena, Germany; ^2^Medical Faculty, Institute of Anatomy, University Duisburg-Essen, Essen, Germany; ^3^Infection Biology, Leibniz Institute for Natural Product Research and Infection Biology, Hans Knöll Institute (HKI), Jena, Germany; ^4^Institute of Microbiology, Friedrich Schiller University Jena, Jena, Germany; ^5^Research Group Microbial Immunology, Leibniz Institute for Natural Product Research and Infection Biology, Hans Knöll Institute (HKI), Jena, Germany; ^6^Center for Sepsis Control and Care (CSCC), University Hospital Jena, Jena, Germany

**Keywords:** carcinoembryonic antigen-related cell adhesion molecule 1, *Candida albicans*, colonization, dissemination, candidiasis mouse model, bone marrow-derived neutrophils, internal transcribed spacer sequencing, mycobiota

## Abstract

Carcinoembryonic antigen-related cell adhesion molecule 1 (CEACAM1, CD66a) is a receptor for *Candida albicans.* It is crucial for the immune response of intestinal epithelial cells to this opportunistic pathogen. Moreover, CEACAM1 is of importance for the mucosal colonization by different bacterial pathogens. We therefore studied the influence of the human CEACAM1 receptor in human CEACAM1-transgenic mice on the *C. albicans* colonization and infection utilizing a colonization/dissemination and a systemic infection mouse model. Our results showed no alterations in the host response between the transgenic mice and the wild-type littermates to the *C. albicans* infections. Both mouse strains showed comparable *C. albicans* colonization and mycobiota, similar fungal burdens in various organs, and a similar survival in the systemic infection model. Interestingly, some of the mice treated with anti-bacterial antibiotics (to prepare them for *C. albicans* colonization *via* oral infection) also showed a strong reduction in endogenous fungi instead of the normally observed increase in fungal numbers. This was independent of the expression of human CEACAM1. In the systemic infection model, the human CEACAM1 expression was differentially regulated in the kidneys and livers of *Candida*-infected transgenic mice. Notably, in the kidneys, a total loss of the largest human CEACAM1 isoform was observed. However, the overwhelming immune response induced in the systemic infection model likely covered any CEACAM1-specific effects in the transgenic animals. *In vitro* studies using bone marrow-derived neutrophils from both mouse strains also revealed no differences in their reaction to *C. albicans*. In conclusion, in contrast to bacterial pathogens interacting with CEACAM1 on different mucosal surfaces, the human CEACAM1-transgenic mice did not reveal a role of human CEACAM1 in the *in vivo* candidiasis models used here. Further studies and different approaches will be needed to reveal a putative role of CEACAM1 in the host response to *C. albicans*.

## Introduction

Carcinoembryonic antigen-related cell adhesion molecule 1 (CEACAM1) is recognized as an important immuno-regulatory receptor in the host response to infections with bacteria that are colonizing human mucosal surfaces such as *Helicobacter pylori*, *Neisseria meningitidis*, *Neisseria gonorrhoeae*, *Moraxella catarrhalis*, and *Fusobacterium* spp. (Gray-Owen and Blumberg, 2006; Slevogt et al., 2008; Klaile et al., 2013; Tchoupa et al., 2014; Javaheri et al., 2016; Horst et al., 2018a; Helfrich and Singer, 2019). CEACAM1 is expressed on epithelial and endothelial cells, as well as on various immune cell types, including neutrophils, monocytes, dendritic cells, NK cells, T cells, and B cells (Gray-Owen and Blumberg, 2006). The highly conserved N-terminal variable Ig-like domain is recognized in a species-specific manner by bacterial, fungal, and viral pathogens (Gray-Owen and Blumberg, 2006; Klaile et al., 2017; Horst et al., 2018a; Helfrich and Singer, 2019). The synchronous engagement of CEACAM1 and other immune receptors, e.g., Toll-like receptors 2 and 4 (Slevogt et al., 2008; Lu et al., 2012; Singer et al., 2014; Schirbel et al., 2019; Zhang et al., 2019) or the inside-out activation of different integrin receptors (Müller et al., 2005; Skubitz and Skubitz, 2008; Muenzner et al., 2010, 2016), results in an altered regulation of the immune response that also depends on the cell type analyzed. Pathogen-CEACAM interactions and the resulting CEACAM1-mediated regulation of immune receptors are not restricted to immune cells but are an important factor in the bacterial colonization of mucosa with regard to the pathogen adherence and the downregulation of the immune response toward the pathogens (Muenzner et al., 2010; Johswich et al., 2013; Islam et al., 2018).

Alternative splicing of the human CEACAM1 mRNA produces different isoforms of which the four major isoforms encompass a long or a short cytoplasmic domain, a transmembrane domain, and either three or four extracellular Ig-like domains (Dankner et al., 2017; Horst et al., 2018a; Helfrich and Singer, 2019). The long cytoplasmic domain comprises two immuno-receptor tyrosine receptor-based inhibition motifs (ITIM) that allow an isoform-specific signal transduction (Gray-Owen and Blumberg, 2006). Especially the attenuating effects of CEACAM1 on the immune cell functions are mediated by CEACAM1 isoforms comprising the ITIM/ITSM motifs in their cytoplasmic domains, and the short isoforms can actually have opposing, immune stimulatory effects (Chen et al., 2004, 2012). While lacking the tyrosine-containing motifs, the short isoforms have several serine phosphorylation motifs, and the ratio between long and short isoforms is known to affect cellular responses regulated by CEACAM1 (Singer et al., 2002; Müller et al., 2009; Dankner et al., 2017; Horst et al., 2018a; Helfrich and Singer, 2019).

We recently found that the opportunistic fungal pathogens *Candida albicans* and *Candida glabrata* bind to human CEACAM1 but not to mouse CEACAM1 (Klaile et al., 2017). In an intestinal epithelial cell model, human CEACAM1 is crucial to the *C. albicans*-induced IL-8 release and the increase in trans-epithelial electrical resistance (Klaile et al., 2017). The gastro-intestinal tracts of up to 80% of critically ill patients are colonized by *Candida* spp., and 5–10% of those patients are diagnosed with invasive candidiasis (Eggimann and Pittet, 2014; Eggimann et al., 2015). Ambivalently, advances in modern medicine led to an increase in the incidence of systemic candidiasis over the past decades, since they raised the numbers of patients with high risk factors, i.e., critically ill and immunosuppressed patients (Eggimann and Pittet, 2014; Lionakis, 2014). Systemic candidiasis is often diagnosed and treated at a late time point, resulting in a high mortality rate that exceeds 30–40% (Lionakis, 2014; Eggimann et al., 2015). Systemic candidiasis often occurs in immunosuppressed patients that are subjected to antibiotic pre-exposure, chemotherapy, and/or hematopoietic stem cell transplantation as a consequence of *C. albicans* dissemination from the gastrointestinal tract (Lionakis, 2014). A second important pathophysiologic origin of systemic candidiasis is the entrance of the pathogen into the bloodstream *via* central venous catheters that compromises the integrity of the cutaneous layer (Lionakis, 2014).

*C. albicans* is an important commensal of the fungal community in the gut (Tso et al., 2018; Bertolini et al., 2019). The mycobiota coexists with the bacterial microbiota and substantially expands the repertoire of organisms interacting with the intestinal immune system to influence health and disease (Iliev et al., 2012; Sam et al., 2017; Krüger et al., 2019). The microbiota can be altered by *C. albicans* and in turn can influence the virulence of the fungal pathogen (Tso et al., 2018; Bertolini et al., 2019). Another study showed that a single receptor may influence this fragile equilibrium between the host and the microbiota: Mice lacking Dectin-1, beside CEACAM1 another important *C. albicans* receptor, exhibited increased susceptibility to chemically induce colitis, due to altered host responses to indigenous fungi (Iliev et al., 2012).

Since CEACAM1 is expressed on intestinal epithelial cells as a part of the enteric mucosal barrier as well as on immune cells like granulocytes that are crucial for the fast innate immune response targeting this pathogen, we hypothesized that the interaction of *C. albicans* with CEACAM1 as immuno-modulatory receptor might be of importance in the pathogenesis of *C. albicans* dissemination from the gut and the induced immune response. In this study, we therefore analyzed the influence of human CEACAM1 expression on the host immune reaction to *C. albicans* in a *C. albicans* colonization and dissemination mouse model mimicking a gastrointestinal tract-derived systemic candidiasis (Koh et al., 2008) and in a systemic model of candidiasis (Jacobsen et al., 2014; Hebecker et al., 2016). For these studies, we used human CEACAM1-transgenic mice that display human CEACAM1 expression in the expected pattern, e.g., on neutrophils and intestinal epithelial cells, as well as in the kidneys and the liver (Gu et al., 2010).

## Materials and Methods

### Microbial Strains and Culture

If not stated otherwise, *Candida albicans* Berkhout strain SC5314 (Gillum et al., 1984) was used for all experiments. For pull-down experiments, also *C. albicans* strain C28a/ATCC 10231 (Bowman et al., 1971), *C. glabrata* strain ATCC 2001 (Clark-Walker et al., 1980), and *Saccharomyces cerevisiae* strain BY4741 (ATCC 4006913) (Winzeler et al., 1999) were applied. All yeasts were a kind gift from Bernhard Hube (Leibniz Institute for Natural Product Research and Infection Biology Hans Knöll Institute, Jena, Germany) and were grown as described below for *Candida albicans*.

*C. albicans* was grown as described (Klassert et al., 2014, 2017). For experiments with BMNs, YPD liquid cultures were inoculated with a single colony from YPD agar plates and grown at 30°C and 180–200 rpm for 14–16 h. Yeast cells were harvested, washed twice, and suspended in a desired volume of ice-cold PBS. Yeast cells were counted in a Neubauer chamber. In some cases, germ tubes were induced by growing 1:100 dilutions of over-night cultures in pre-warmed RPMI at 37°C and 180 rpm for approximately 2 h. Germ tube formation was observed at short intervals microscopically, and further growth/elongation was stopped at a tube length of approximately 1–2 yeast cell diameter(s) by washing germ tubes twice with ice-cold PBS. For some experiments, *C. albicans* yeast cells or germ tubes were UV inactivated by applying 20 J/cm. Inactivated cells were again washed twice in ice-cold PBS and counted. All centrifugation steps were performed at 3,000 × *g*, 4°C for 3 min.

For mouse experiments, *C. albicans* was grown as follows: At day −6, a fresh YPD plate was inoculated from a frozen stock, cultivated at 30°C for 48 h, and kept at 4°C. At day −2, a single colony was sub-cultured on a fresh YPD plate and 30°C for 24 h and a single colony from that plate was used to inoculate 25 ml YPD liquid medium at day −1. The liquid culture was cultivated at 30°C and 180 rpm for 14–16 h. Yeast cells were pelleted, washed twice with 50 ml ice-cold PBS, re-suspended in 15 ml PBS, counted, and kept on ice. Negative Gram stain verified the absence of contaminating bacteria. Yeast cells were adjusted to the desired concentration. All centrifugation steps were performed at 3,000 × *g*, 4°C for 3 min. *C. albicans* solutions were warmed to 30°C immediately before oral gavage or injection. CFU counts were verified by plating dilutions before and after infections on YPD agar plates.

*Moraxella catarrhalis* BBH18 bacteria (kindly provided by Kristian Riesbeck, Malmö Lund University, Skåne University Hospital, Malmö, Sweden) (De Vries et al., 2010) were grown over night at 37°C, 5% CO_2_ on Columbia agar plates (BD Biosciences) (Heinrich et al., 2016). About 15 ml of BHI broth (Brain Heart Infusion, BD Biosciences) was inoculated to an optical density (OD)_600 nm_ of 0.03–0.06 and incubated at 37°C, 180 rpm until an OD_600 nm_ between 0.3 and 0.6 was reached. Bacterial cells were harvested, washed twice, and homogenized in a desired volume of in ice-cold PBS. The OD_600 nm_ was determined and used to calculate the concentration of bacteria (OD_600 nm_ = 0.3 = 5 × 10^7^ CFU/ml).

### FITC Labeling of *C. albicans* Yeast Cells

*C. albicans* yeast cells from an overnight culture grown as described above were washed once with PBS, and the concentration was determined using a Neubauer chamber. Up to 200 μl yeast cell pellet was suspended in 10 ml carbonate buffer (pH 9.5; 70% of sodium bicarbonate, 30% of sodium carbonate), and the concentration was adjusted to 1 × 10^7^ yeast cells per ml carbonate buffer. About 100 μl of a FITC stock solution (10 mg/ml in PBS) was added to 10 ml yeast solution to result in a final concentration of 0.1 mg/ml FITC/PBS and incubated for 1 h at 23°C, 150 rpm in the dark. Labeled yeast cells were washed twice with PBS and suspended at 5 × 10^7^ yeast cells/ml in RPMI/10% FCS.

### Mouse Strains

FVB mice transgenic for the human CEACAM1 gene (Gu et al., 2010) were crossed into the C57BL/6N background using Speed Congenics. The status of the background was supervised by GVG Genetic Monitoring (Leipzig, Germany). Mice were bred heterozygous, and a minimum of 12 backcrosses were performed prior to the first experiment. The genotypes (CEACAM1± or wild type) were determined by polymerase chain reaction (PCR) analysis of tail biopsies using the following primer pair for the transgenic human CEACAM1: CCACTTCACAGAGTGCGTGT + ATTGTCTCTCGACCGCTGTT, and mouse interferon beta primers ATAAGCAGCTCCAGCTCCAA + GCAACCACCACTCATTCTGA as positive control. Wild-type littermates were co-housed and used as controls. Mice were maintained under specific pathogen-free conditions at the animal facility *Forschungszentrum Beutenberg, Zentralen Experimentellen Tierhaltung*, University Hospital Jena, Germany, according to European and German animal welfare regulations.

### Ethics Statement

Animal studies were performed in strict accordance with European (The Council of Europe’s European Convention, March 18, 1986, relating to the protection of animals used for experimental and other specific purposes with the revised Annex A 2010/63/EU, June 18, 2007, to this Convention on guidelines for the accommodation and care of animals used for experimental and other scientific purposes; The European Parliament and Council Directive 2010/63/EU d 22.09.2010 regarding the protection of animals used for experimental and other scientific purposes that came into force January 01, 2013) and German animal welfare regulations and the recommendations of the Society for Laboratory Animal Science (GV-Solas). All experiments were approved by the ethics committee “Beratende Kommission nach §15 Abs. 1 Tierschutzgesetz” and the responsible Federal State authority *Thüringer Landesamt für Verbraucherschutz*, Bad Langensalza, Germany (Permit No. 02-019/14).

### Colonization/Dissemination Model

Table 1 gives an overview of this murine model of antibiotic-induced GI tract colonization by *C. albicans* and immunosuppressive-induced dissemination based on the approach established by Koh et al. (2008) with slight modifications. Power and mouse numbers were calculated according to Jacob (1977) by one-way ANOVA. Calculations for the colonization/dissemination model revealed that for the analysis of fungal burdens with an effective power of 0.8 (type 1 error = 0.05/type 2 error = 0.2), five animals per group are sufficient. Since after the first experiment no difference at all was observed for the main effect sizes, we decided because of animal welfare not to repeat the experiment to prove reproducibility. About 10–12-week-old CEACAM1-transgenic mice (*N* = 5) and their wild-type littermates (*N* = 5) were co-housed as groups of five in two sterilized, individually ventilated cages supplied with sterile bedding, sterile enrichment, sterile water, and sterile mouse chow. Mice were adapted to sucrose in their drinking water (2.5% sucrose for 2 days and 5% sucrose for 1 day). To deplete the indigenous bacterial flora and to allow colonization by *C. albicans*, mice were fed sterile water with 2 mg/ml streptomycin, 1,500 U/ml penicillin, 7.5% sucrose (to achieve a better acceptance of the water), and 625 mg/kg doxycycline in the chow (TD.01306, Rodent diet = 2018 base diet + 625 mg/kg doxycycline; Harlan Laboratories GmbH; Maasheseweg 87/C, 5804 AB Venray) for 4 days before the oral *C. albicans* inoculation (day −4) until the end of the experiment (day 14). Mice were inoculated with 5 × 10^7^ colony forming units (CFUs) *C. albicans* SC5314 yeast cells (grown as described above) *via* oral gavage to start colonization at day 0. At day 11, mice were injected intraperitoneal with 150 mg/kg cyclophosphamide monohydrate (Endoxan; Baxter Oncology GmbH, Germany) to induce *C. albicans* dissemination. Mice were monitored and scored according to the protocol given in section “Systemic *C. albicans* Infection Model.”

**Table 1:** Overview of treatments for the colonization/dissemination study.

Day	Treatment	Analysis
−7	Start addition of sucrose to drinking water	
−4	Start **antibiosis** (water)	CFU: feces ITS/16S qPCR: feces ITS sequencing: feces
0	Oral gavage with *C. albicans* = **begin of colonization**	CFU: feces ITS/16S qPCR: feces ITS sequencing: feces
2		CFU: feces
4		CFU: feces
7		CFU: feces
11	Injection of cyclophosphamide = **induction of dissemination**	CFU: feces ITS/16S qPCR: feces ITS sequencing: feces
14	**Necropsy**	CFU: feces, blood, kidney, liver, spleen, brain, small intestines, cecum, colon, intestinal lymph nodes, small intestine contents, cecum contents, and colon contents

Stool was collected from individual mice at day −4 (before antibiosis), day 0 (before *C. albicans* inoculation), and days 2, 4, 7, and 11 (before cyclophosphamide injection) and at day 14 (end of experiment). Two fecal pellets were weighed and kept on ice until homogenization in 1 ml PBS for the determination of the CFU counts by plating dilutions on yeast extract-peptone-dextrose (YPD) agar plates with and/or without 80 μg/ml chloramphenicol. On days −4, 0, and 11, residual pellets were weighed, snap-frozen in liquid nitrogen, and stored at −80°C for analysis of mycobiota (protocols below). Mice were sacrificed at day 14 by intraperitoneal injection of 500 mg/kg ketamine and 50 mg/kg xylazine (both MEDISTAR Arzneimittelvertrieb GmbH, Germany), followed by collection of peripheral blood from the vena cava. About 100 μl of the peripheral blood was plated undiluted on YPD agar plates with 80 μg/ml chloramphenicol in order to determine CFU counts. During necropsy, kidneys, liver, spleen, small intestines, cecum, colon, intestinal lymph nodes, and brain were removed and weighed. Contents from small intestines, cecum, and colon were obtained by careful rinsing with PBS (3, 2, and 2 ml, respectively) and kneading; washed small intestines, cecum, and colon were weighed again. Intestinal contents and all organs were kept on ice in 1–3 ml PBS until homogenization. Intestinal contents were homogenized by vigorous pipetting. Organs were homogenized aseptically with a T 10 basic ULTRA-TURRAX disperser. CFUs were determined by plating dilutions on YPD agar plates with 80 μg/ml chloramphenicol. The detection limits were as follows: 17 CFU/g feces; 50 CFU/g kidney, liver, intestinal lymph nodes, small intestines, cecum, or colon; 10 CFU/ml blood; 100 CFU/g spleen; 85 CFU/g brain; 90 CFU/g small intestine content; 30 CFU/g cecum content; and 150 CFU/g colon content.

### DNA Extraction From Feces

DNA extraction from frozen feces was performed using the innuPREP Stool DNA extraction kit with a 5-min homogenization step in a SpeedMill (both Analytik Jena AG). DNA concentrations were determined using a Qubit Fluorometer (Thermo Fisher Scientific).

### RNA Extraction and cDNA Synthesis

RNA extraction from mouse livers and kidneys was performed using the innuPREP RNA Mini Kit (Analytik Jena AG) according to the manufacturer’s instructions for tissue samples. RNA concentrations were determined on a NanoDrop D-1000 Spectrophotometer (Thermo-Fisher Scientific). First-strand complementary DNA (cDNA) was synthesized from 1 μg of RNA using the High Capacity cDNA Reverse Transcription Kit (Life Technologies GmbH).

### Real Time Polymerase Chain Reaction Analysis of Human CEACAM1 Isoforms

Real time (RT) PCR analysis of human CEACAM1 isoforms was performed as described in detail by Klaile et al. (2013) using the following primer pairs (all sequences 5′–3′ followed by the expected product size in brackets): CEACAM1-4L: AAGACGATCATAGTCACTGAGCT + GGAGACTGAGGGTTTGTGCT (483 bp); CEACAM1-4S: AAGACGATCATAGTCACTGAGCT + ATTGGAGTGGTCCTGAGCTG (454 bp); CEACAM1-3L: AGACGATCATAGTCACTGATAATGC + GGAGACTGAGGGTTTGTGCT (188 bp); CEACAM1-3S: AGACGATCATAGTCACTGATAATGC + ATTGGAGTGGTCCTGAGCTG (159 bp); CEACAM1-4C1: AAGACGATCATAGTCACTGAGCT + TTGCACACCATTGACAGAGT (369 bp); CEACAM1-3: CAGTGACCCAGTCACCTTGA + TGGACTTGTTTGTGCCTGTTG (403 bp); CEACAM1-3C2: CAAGACGATCATAGTCACTGAGTC + AGAGGGACATATAGGAAGGGGT (210 bp); panCEACAM1 (amplify all human CEACAM1 isoforms bearing exons 4 and 9): CAGGACCACAGTCAAGACGA + GGTTGCTGGGCTTCAAAGTT (CC1-4L = 582 bp; CC1-4S = 529 bp; CC1-3AL = 387 bp; CC1-3AS = 333 bp; CC1-3L = 294 bp; CC1-3S = 240 bp). HPRT1 served as positive control: GACCAGTCAACAGGGGACAT + AACACTTCGTGGGGTCCTTTTC (195 bp). Products were separated on an agarose gel containing GelRed Nucleic Acid Gel Stain (Biotium) and visualized under a UV transilluminator.

### Quantitative Polymerase Chain Reaction Analysis of Internal Transcribed Spacer and 16S Copy Numbers

In order to quantify the internal transcribed spacer (ITS) and 16S copy numbers in DNA extracted from fecal samples, we used a CAS-1200 pipetting robot (Qiagen) to set up the quantitative PCRs (qPCRs) and a Corbett Rotor-Gene 6000 (Qiagen) as real time (RT) qPCR apparatus. Each sample was analyzed in duplicate in a total reaction volume of 20 μl containing 10 μl of 2× SensiMix SYBR Master Mix (Bioline) and 0.2 μM of each primer [ITS: CTTGGTCATTTAGAGGAAGTAA + GCTGCGTTCTTCATCGATGC; 16S: GTGYCAGCMGCCGCGGTAA + GGACTACNVGGGTWTCTAAT, sequences from the Earth Microbiome Project; http://press.igsb.anl.gov/earthmicrobiome/protocols-and-standards/its/ and http://press.igsb.anl.gov/earthmicrobiome/protocols-and-standards/16s/, respectively (Thompson et al., 2017)]. The cycling conditions included an initial step of 95°C for 10 min followed by 40 cycles of 95°C for 15 s, 60°C for 20 s, and 72°C for 20 s. For each experiment, an RT-negative sample and 16S and ITS standards were included. Melting curve analysis and size verification by electrophoresis were used to confirm the specificity of the qPCRs. The relative expression of the target genes was analyzed using 16S and ITS standards. Note that copy numbers for both genes vary within the kingdoms; bacteria can have more than 10 copies of the 16S region (Vetrovsky and Baldrian, 2013; Louca et al., 2018), and fungi can have more than 100 copies of the ITS region (Maleszka and Clark-Walker, 1993; Lofgren et al., 2019).

### Internal Transcribed Spacer and 16S DNA Sequencing

Extracted DNA was used as input for PCR amplification of the ITS region of the rDNA. The Illumina ITS Primer Constructs (ITS1f-ITS2) for the amplification were fused with Golay indices and adapter sequences as described in the Earth Microbiome Project protocol (Thompson et al., 2017)^1^. The PCR (94°C, 3 min/35 × 94°C, 30 s; 52°C, 30 s; 72°C, 60 s/72°C, 10 min) was performed on a S1000™ Thermal Cycler (BIORAD) in 50 μl reactions using the Platinum™ PCR SuperMix (Thermo Fisher Scientific). After purification using NucleoMag NGS Clean-up and Size Select (Macherey-Nagel), size distributions and concentrations of the PCR products were analyzed on a TapeStation 2200 (Agilent Technologies). Final libraries were pooled in equimolar amounts and prepared for Illumina Sequencing using the MiSeq Reagent Kit v2 (Illumina) following manufacturers’ instructions. Run plan and reagents were adapted according to Caporaso et al. (2012). Sequencing was performed on a MiSeq apparatus (Illumina) with 251 cycles.

### Analysis of Internal Transcribed Spacer DNA Sequences

The resulting fastq files were quality checked using FastQC, v0.11.5 (Andrews, 2010), trimmed with Trimmomatic v0.36 (Bolger et al., 2014), and forward and reverse reads merged using PEAR v0.9.10 (Zhang et al., 2014). All assembled sequences and all remaining forward reads that could not be merged were combined (median number of sequences per sample: 118,535) and further analyzed using QIIME v1.9.1 (Caporaso et al., 2010). Thereafter, open reference OTU picking was performed against UNITE (Tang et al., 2015)^2^. Taxonomies for each of the samples were summarized, and the alpha and beta diversities (Bray-Curtis distance) were calculated. Analysis of similarity (ANOSIM) was performed by comparing the Bray-Curtis distances using days, genotypes, and cages as categorical variables.

### Systemic *C. albicans* Infection Model

The infection was performed as described previously (Jacobsen et al., 2014; Hebecker et al., 2016). Power and mouse numbers were calculated according to Jacob (1977) by one-way ANOVA and show that 16 mice per group are sufficient to detect differences in survival times of ±1 day with an effective power of 0.8 (type 1 error = 0.05/type 2 error = 0.2). Briefly, 10–12-week-old CEACAM1-transgenic mice and their wild-type littermates were co-housed as groups of maximal five animals in sterilized, individually ventilated cages supplied with sterile bedding, sterile enrichment, sterile water, and sterile mouse chow*. C. albicans* was prepared as described above. On day 0, mice were infected with 2.5 × 10^5^ CFU *C. albicans*/g body weight (corresponding to a total of 5 × 10^6^ CFU *C. albicans* for a mouse of 20 g body weight) or 1 × 10^5^ CFU *C. albicans*/g body weight (corresponding to a total of 2 × 10^6^ CFU *C. albicans* for a mouse of 20 g body weight) *via* the lateral tail vein. After infection, the health status of the mice was examined at least twice a day, and surface temperature and body weight were recorded daily. The general condition and behavior were evaluated and documented according to the following scored parameters that were chosen according to typical symptoms occurring in a systemic *Candida albicans* infection in mouse models: (1) body weight: no difference/reduction <10%/day = score 0, reduction >10%/day = score 1, and reduction >20% total = score 2; (2) general condition: smooth, glossy hair coat = score 0, slightly ruffled hair coat = score 1, and ruffled hair coat = score 2; (3) behavior: spontaneous activity/normal = score 0, reduced spontaneous activity = score 1, little spontaneous activity/slightly delayed reaction to external stimuli = score 2, and delayed/no reaction to external stimuli = score 3; and (4) body temperature: normal = score 0, moderately increased (≥ + 1.0°C but ≤ + 2°C) = score 1, considerably increased (> + 2.0°C) or moderately decreased (≤ − 1.5°C) = score 2, and considerably decreased (> − 1.5°C) = score 3. A humane endpoint was defined as a score of 3 in one category or a total (additive) score of 5. Mice were sacrificed by intraperitoneal injection of 500 mg/kg ketamine and 50 mg/kg xylazine (both MEDISTAR Arzneimittelvertrieb GmbH, Germany) when reaching a humane endpoint. When mice reached deep anesthesia, blood was taken retro-orbital and analyzed in a hemocytometer. Kidneys, spleen, liver, and brain were removed, weighed, and kept on ice in 1–3 ml PBS until homogenization. Organs were homogenized with a T 10 basic ULTRA-TURRAX disperser. CFUs were determined by plating dilutions on YPD agar plates with 80 μg/ml chloramphenicol. Survival data are combined from two independent experiments and groups included 12 (WT, high infection dose) or 16 animals (CC1, both infection doses, and WT low infection dose). For the Log Rank comparison of the survival curves with the GraphPad PRISM 5 software, the surviving animal was censored. The Kaplan Meier analysis of survival rates includes the surviving mouse. Data are pooled from two independent experiments. Three CEACAM1-transgenic mice and three wild-type mice were sacrificed without any prior treatment by cervical dislocation. Organs were removed and treated as described above for the systemic infection model.

### Western Blot Analysis of Organ Samples

About 500 μl aliquots of the homogenized kidney and liver samples were mixed with 100 μl 6 × lysis buffer (PBS/6 × complete EDTA-free protease inhibitor cocktail, Sigma Aldrich GmbH/6% TX100/0.6% SDS/6 mM phenylmethanesulfonyl fluoride) and kept on ice for 1 h. Samples were cleared at 16,000 × *g*, 4°C, for 15 min, mixed with ¼ volume 5 × Laemmli buffer [14.5% SDS (w/v)/0.3 M Tris-HCl pH6.8/50% Glycerol/0.015% brome phenol blue (w/v)], and heated to 95°C for 5 min. Samples were run on 15-well Mini-Protean TGX Gels 4–15% (Bio-Rad Laboratories GmbH) at 50 V (constant) and transferred to nitrocellulose membranes (Thermo Scientific) at 280 mA (constant) for 2 h using the Mini Trans Blot Cell system (Bio-Rad Laboratories GmbH). Membranes were blocked with PBS/10% skim milk powder for at least 1 h and developed using C5-1X (101-M181Reliatech GmbH) and 20-33 (anti-actin, Sigma Aldrich GmbH; loading control) antibodies and HRP-conjugated goat anti-mouse IgG (115-035-166, Dianova GmbH) or goat anti-rabbit IgG (111-035-144, Dianova GmbH), respectively. Signals were detected using SuperSignal West Pico Chemiluminescent Substrate (Fisher Scientific GmbH) with a Fusion FX7 Imager (PEQLAB Biotechnologie GmbH). Images were processed with Adobe Photoshop CS5 (Adobe).

### Isolation of Bone Marrow-Derived Neutrophils

Eight- to twelve-week-old mice were sacrificed by cervical dislocation. Femurs and tibias were cleaned from tissue, sprayed with 70% ethanol, and stored in 1 × PBS on ice. Both ends of the bones were cut, and bone marrow cells were collected by injection of PBS and filtered through a cell strainer (40 μm). The bone marrow cell suspension was centrifuged at 250 × *g* for 5 min at room temperature, and the pellet was re-suspended in 3 ml erythrocyte lysis buffer (Ammonium-Chloride-Potassium Lysing Buffer, Thermo Fisher Scientific). After incubation for 2 min at room temperature, 7 ml PBS was added before centrifugation, and pellets were washed once in 10 ml PBS. BMNs were then separated by density gradient centrifugation. In a 15 ml Falcon tube, 3 ml pre-warmed (room temperature) Histopaque 1,119 was carefully overlaid with 3 ml Histopaque 1,077 (both Sigma-Aldrich GmbH) and 1 ml bone marrow cell suspension and centrifuged at 830 × *g*, RT with slow acceleration and without brake for 30 min. BMNs were collected at the interface of the Histopaque 1,119 and 1,077 layers and washed twice with RPMI 1,640/10% FBS (300 × *g*, 7 min, room temperature). Purity and viability of BMNs were assessed on an Attune Acoustic Focusing Cytometer (Thermo Fisher Scientific) using the Attune software v2.1. All assays with BMNs were performed in Eppendorf tubes blocked with 10% BSA/PBS, for at least 1 h at 37°C. If not stated otherwise, all centrifugation steps were performed at 280 × *g* and 4°C for 5 min. Directly after the isolation granulocytes were stained with eFluor780 viability dye (eBiosciences) and analyzed by flow cytometry for purity (above 80%) and viability (above 96%). Cell numbers were adjusted according to viable granulocyte counts for the individual experiments.

### Phagocytosis Assay

Phagocytosis assays were performed with freshly prepared BMNs. Eight-well chamber slides were coated with 500 μl 0.001% Poly-L-Lysine for at least 1 h and dried under the safety cabinet. A 1 × 10^6^ BMNs in 500 μl RPMI/10% FCS were seeded per well and incubated for 30–60 min at 37°C, 5% CO_2_ in order to allow the BMNs to adhere. FITC-labeled *C. albicans* yeast cells (prepared as described above) were adjusted to a concentration of 2 × 10^8^ cells/ml. Before infection, 200 μl medium was removed from each well, and 100 μl FITC-labeled *C. albicans* cells were added (MOI = 20). The chamber slide was centrifuged at 100 × *g* for 5 min and incubated for 20 min at 37°C, 5% CO_2_. Wells were fixed with 500 μl of 4% paraformaldehyde/PBS per well for 20–40 min at room temperature and blocked overnight with 500 μl 5% BSA/PBS at 4°C. Samples were then incubated with mouse Fc block (Miltenyi Biotec GmbH) for 30 min at 4°C, incubated with anti-mouse CEACAM1 antibody (MSCC1, monoclonal mouse IgG1, Bernhard B. Singer, Essen) and anti-*Candida albicans* antibody (polyclonal rabbit IgG, Acris Antibodies Germany; #BP1006) at 0.35 μg/100 μl 5% BSA/PBS for 1 h, washed, and incubated with secondary staining mix (1:3,000 Hoechst 33342, 1:200 goat anti-mouse-Alexa546, and 1:200 goat anti-rabbit-Alexa633 in 5% BSA/PBS) for 2 h. The slide was washed and mounted with Vectashield mounting medium (Biozol Diagnostica Vertrieb GmbH). Samples were analyzed with a confocal laser scanning microscope (Zeiss LSM 710) using the ZEN 2010 software (both Carl Zeiss Microscopy GmbH). Micrographs were analyzed for phagocytosis by counting BMNs without contact to *Candida* cells, BMNs with attached, extracellular *Candida* cells (FITC staining and anti-*Candida* antibody staining), and BMNs with intracellular (phagocytosed) *Candida* cells (FITC staining only).

### Killing Assay

Overall candidacidal activity of BMNs was tested with freshly prepared BMNs in RPMI/10% FBS by incubating 2.5 × 10^5^ BMNs with 2.5 × 10^4^ *Candida* yeast cells (MOI 0.1) for 60 min. BMNs were lysed with the addition of Triton X-100 to a final concentration of 0.25%, and surviving *Candida* cells were counted after plating different dilutions on YPD agar. Yeast cells without the presence of neutrophils served as controls.

### Flow Cytometry

Analysis of relative fluorescence intensities was performed on an Attune Acoustic Focusing Cytometer (Thermo Fisher Scientific) using the Attune software v2.1. The expression of CD11b and mouse and human CEACAM1 in BMNs was determined in cells either left untreated or stimulated with UV-killed *C. albicans* germ tubes (MOI 10) for 60 min. Cells were stained using viability dye eFluor780 (eBiosciences) and humanized antibodies directly conjugated to fluorescent dyes (all Miltenyi Biotec): anti-CD11b-ViobrightFITC (clone REA592), anti-human CD66acde-PE (clone REA428; recognizes human CEACAM1), and anti-mouse CD66a (clone REA410; mouse CEACAM1 specific).

For analysis of apoptosis, BMNs were either left untreated for 0 or 5 h or treated for 5 h with 50 ng/ml PMA or with UV-killed *C. albicans* germ tubes (MOI 10) and stained using the Annexin V Detection Kit APC (eBiosciences).

For analysis of intracellular reactive oxygen species, BMNs were pre-incubated for 15 min with 1.5 μg/ml dihydrorhodamine 123 (DHR) fluorigenic probe (Biomol GmbH) in calcium- and magnesium-free PBS/2.5% BSA and either left untreated or stimulated with UV-killed *C. albicans* germ tubes (MOI 10) for another 15 min. Cells were washed in calcium- and magnesium-free PBS, fixed in 1% PFA/PBS for 10 min, blocked with PBS/50% heat-inactivated fetal bovine serum, and washed with PBS/2% heat-inactivated fetal bovine serum before analysis.

### Myeloperoxidase Enzyme-Linked Immunosorbent Assay

Concentrations of released MPO were determined in cell culture supernatants from BMNs that were either left untreated or stimulated with live *C. albicans* yeast cells (MOI 10) for 60 min using the mouse MPO enzyme-linked immunosorbent assay (ELISA) kit from Hycultec GmbH.

### Gelatinase Activity Assay

MMP9 (gelatinase) activity was determined in cell culture supernatants from BMNs that were either left untreated or stimulated with 50 ng/ml PMA or *C. albicans* yeast cells (MOI 10) for 60 min by measuring the relative fluorescence intensity induced by the degradation of the EnzCheck DQ-Gelatin-substrate (75 μg/ml, 24 h) in an INFINITE M200 instrument (Tecan; excitation 495 nm; emission 515 nm).

### Pulldown Assays

Recombinant human CEACAM1 protein consisting of the CEACAM1 extracellular domain fused to the constant region of human IgG was produced in HEK-293 cells and purified *via* protein G columns (GE Healthcare, Munich, Germany) as described previously (Klaile et al., 2009). Recombinant mouse CEACAM1-His was purchased from Hölzel Diagnostika GmbH. Pulldown assays were performed with 2 × 10^8^ live yeast cells (*C. albicans* SC5314, *C. albicans* 28a, *C. glabrata* 2001, and *Saccharomyces cerevisiae* BY4741) or *M. catarrhalis* cells and 1 μg recombinant protein. Before the incubation with fungal/bacterial cells, each recombinant protein was pre-treated with the addition of at least 1 volume of 100 mM glycine (pH 2.2) in order to break up potential CEACAM1 homo-dimers. After 10 min, the pH was restored with the addition of 1 volume 1 M Tris-HCl (pH 8.0). Fungal/bacterial cells were incubated for 2 h with the recombinant proteins, washed twice with PBS, and eluted with 40 μl 100 mM glycine (pH 2.2). Supernatants and eluates were analyzed by Western blotting performed as described above for the presence of the respective recombinant protein. Proteins were detected using HRP-conjugated goat anti-human IgG Fc antibody or MSCC1 (anti-mouse CEACAM1; mouse IgG1, Bernhard B Singer, Essen) and HRP-conjugated goat-anti mouse IgG (115-035-166, Dianova GmbH), respectively. Signals were detected using SuperSignal West Pico Chemiluminescent Substrate (Fisher Scientific GmbH) with a Fusion FX7 Imager (PEQLAB Biotechnologie GmbH). Images were processed with Adobe Photoshop CS5 (Adobe).

### Statistical Analysis

Except for sequencing data, statistical analysis was performed using GraphPad Prism 5.04 Software. For parametric data with two groups, unpaired, two-tailed Student’s *t* test was performed; for matched pairs, a paired two-tailed Student’s *t* test was performed. For non-matched parametric data with more than two groups, one-way ANOVA with Bonferroni post-tests was performed. In case of exponential data (CFUs, relative fluorescence intensity), log(10) transformed data were used for statistical analysis. In case of samples with no detectable CFU counts, statistical analysis was performed twice, inserting either 0.1 or the respective detection limit; the outcome was “not significant” in both cases. In the present manuscript, the values of *p* for the former analysis are given. For the analysis of beta diversities of sequencing data, ANOSIM was performed on Bray-Curtis distances using QIIME.

## Results

### Co-housing but Not Human CEACAM1 Expression Affected the Gastrointestinal Mycobiota After Antibiosis

Since our *in vitro* data obtained from a human intestinal epithelial cell model show that the lack of CEACAM1 prevents a normal mucosal response to *C. albicans* (Klaile et al., 2017), we studied the influence of human CEACAM1 in a transgenic gain-of-function mouse strain (Gu et al., 2010). The CEACAM1-transgenic mice and their wild-type littermates were subjected to a *C. albicans* colonization/dissemination model summarized in Table 1.

The composition of the gut microbiota is an important factor in the colonization and subsequent infection by *C. albicans* (Forster et al., 2016; Charlet et al., 2018; Krüger et al., 2019; Matsuo et al., 2019). Since genetic factors, i.e., the presence or absence of a specific receptor like Dectin-1, NLRP6, or TLR5 can influence the microbiome and also the gut homeostasis and host responses (Iliev et al., 2012; Marietta et al., 2015), we hypothesized that the expression of human CEACAM1 on the intestinal epithelial mucosa may influence their response to fungal ligands and therefore the homeostasis of the gut mycobiota.

**Figure 1:**
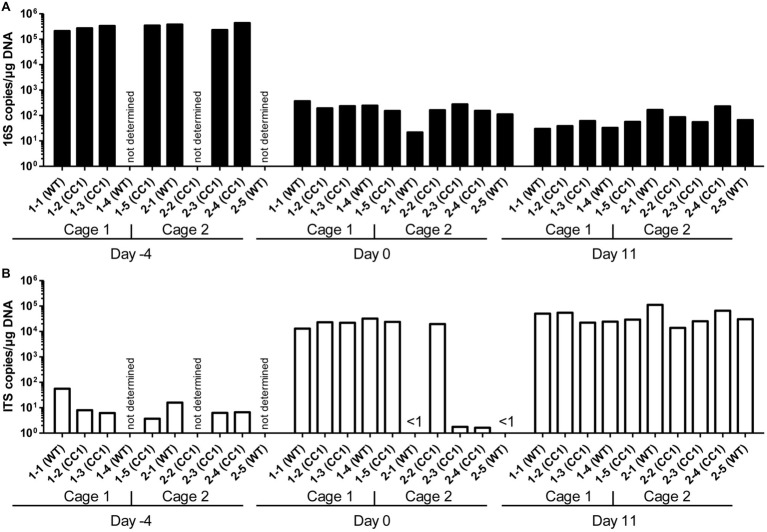
Influence of antibiosis and intestinal colonization by *C. albicans* on the relative abundance of bacteria and fungi. Human CEACAM1-transgenic mice (CC1, *N* = 5) and their wild-type littermates (WT, *N* = 5), co-housed in two cages, were treated with antibiotics from day −4 and inoculated with 5 × 10^7^ CFU *C. albicans* orally at day 0 (see also Table 1). Feces were collected from individual mice on day −4 (untreated/before antibiotic treatment), day 0 (4 days of antibiosis/before *C. albicans* inoculation), and day 11 (11 days of *C. albicans* inoculation, continuous antibiosis). DNA was extracted and analyzed by qPCR for copy numbers of bacterial 16S DNA **(A)** and fungal ITS DNA **(B)**. Copies per μg DNA were calculated by standards included in the qPCR runs. Note that for three samples (labeled as “not determined”) not enough feces was left for this analysis since the samples were necessary to determine the primary effect size (fungal colonization) shown in Figure 3A. All data are from one single experiment.

We first analyzed the influence of the expression of human CEACAM1 on the relative abundance of bacterial and fungal components of the microbiota in the feces by RT-PCR analysis of 16S and ITS copy numbers (Figure 1). Feces collected from individual mice on day −4 (before the start of the antibiosis) revealed an excess of 16S copies/μg DNA over ITS copies/μg DNA of almost four orders of magnitude. Thus, as expected, bacteria by far outnumbered fungi in the mouse feces. The actual gap between cell numbers of both kingdoms is even greater, since fungal species can possess more than 100 ITS copies in their genome, while bacterial genomes contain 1–15 16S copies (Black et al., 2013; Vetrovsky and Baldrian, 2013; Louca et al., 2018; Lofgren et al., 2019).

**Figure 2:**
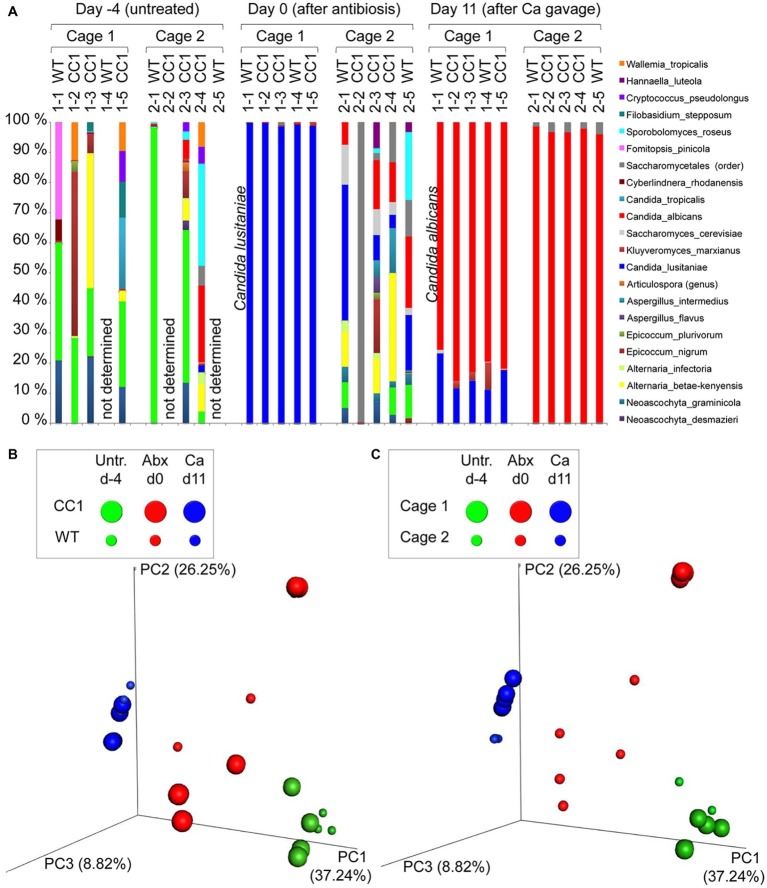
Co-housing, but not the expression of human CEACAM1 influences the fungal beta diversities in a *C. albicans* colonization model. DNA samples studied in Figure 1 were analyzed for fungal species by ITS sequencing. Human CEACAM1-transgenic mice (CC1, *N* = 5) and their wild-type littermates (WT, *N* = 5), co-housed in two cages, were treated with antibiotics from day −4 and inoculated with 5 × 10^7^ CFU *C. albicans* orally at day 0 (see also Table 1). Feces were collected from individual mice on day −4 (untreated = before antibiotic treatment), day 0 (4 days of antibiosis/before *C. albicans* inoculation), and day 11 (11 days of *C. albicans* inoculation, continuous antibiosis). Note that for three samples (labeled as “not determined”) not enough feces was left for this analysis, since the samples were necessary to determine the primary effect size (fungal colonization) shown in Figure 3A. **(A)** Bar plots of operational taxonomic units (OTUs) detected in fecal samples with a minimum relative abundance of 0.1% of all detected OTUs in at least one sample. See also Supplementary Tables S1–3 for a list of the species detected with a minimum occurrence of 0.1% in at least one sample and their relative abundance in all samples. **(B,C)** PCoA plots of calculated Bray-Curtis distances (beta diversities) of fungal species between all samples were generated and subsequently analyzed for the similarity (ANOSIM) between different sample groups: **(B)** WT vs. CC1 and **(C)** cage 1 vs. cage 2 in untreated mice (Untr., day −4), after antibiosis (Abx, day 0), and after oral *C. albicans* inoculation (Ca, day 11). Statistical results of comparisons between groups are given in the text. All data are from one single experiment. (See also Supplementary Figure S1A for alpha diversity/Shannon index.)

After 4 days of antibiosis (day 0), a loss of 16S copies/μg DNA of around three orders of magnitude was detected compared to the average value at day −4, and values remain similar also after the colonization with *C. albicans* under continuous antibiosis (day 11) (Figure 1A). In 6 of 10 mice, ITS copies/μg DNA increase after antibiosis (day 0) around three orders of magnitude compared to the average ITS copy numbers of untreated mice at day −4, while four mice display a near complete loss of their ITS DNA (Figure 1B). Other groups also observed an increase in fungal colonizers after antibiosis in mice (Dollive et al., 2013; Azevedo et al., 2015). The surprising finding of a reduction in ITS copies/μg DNA in four mice is supported by the accompanying loss of fungal colony growth when fecal samples were plated on YPD agar plates (Figure 2A; Supplementary Figure 1). Interestingly, all four mice were from one cage (2 WT and 2 CC1). We can only speculate that the general dysbiosis induced by the antibiotic treatment lead to this reduction in fungal commensals (Krüger et al., 2019). After oral *C. albicans* inoculation, all mice displayed similarly high ITS copies/μg DNA (Figure 1B).

**Figure 3:**
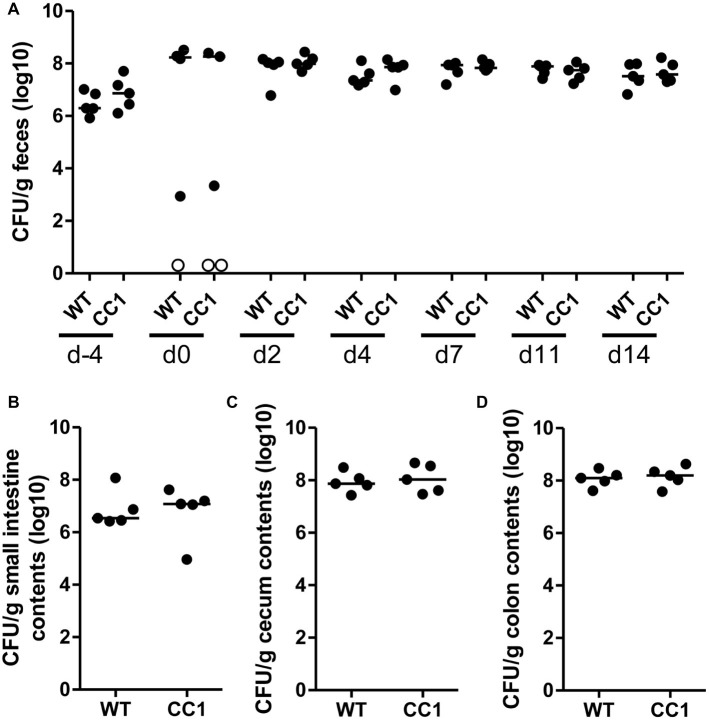
The expression of human CEACAM1 does not influence the intestinal colonization by *C. albicans*. Human CEACAM1-transgenic mice (CC1, *N* = 5) and their wild-type littermates (WT, *N* = 5), co-housed in two cages, were treated with antibiotics from day −4, inoculated with 5 × 10^7^ CFU *C. albicans* orally at day 0 and injected with cyclophosphamide at day 11 (see also Table 1). Feces were collected from individual mice at the days indicated and analyzed for CFU content by plating dilutions on YPD agar **(A)**. Note that similar CFUs were detected on YPD agar containing 80 μg/ml chloramphenicol (Supplementary Figure S1B). Open circles represent samples without any CFUs detected by plating on either YPD plate type. On day 14, mice were sacrificed and contents from small intestine **(B)**, cecum **(C)**, and colon **(D)** were collected by rinsing intestines with PBS and analyzed for their CFU content. Graphs show CFU/g feces (log10) with median. Statistical analysis was performed with logarithmized data by one-way ANOVA and Bonferroni post-test **(A)** or by unpaired, two-sided Student’s *t* tests **(B–D)**; no significant differences were detected between the two genotypes at any time/in all matching contents (**B**: *p* = 0.879, **C**: *p* = 0.671, **D**: *p* = 0.729). All data are from one single experiment.

We next analyzed the influence of the expression of human CEACAM1 on the composition of the mycobiota and identified fungal species in feces *via* ITS1 sequencing at days −4, 0, and 11, i.e., in untreated animals, after antibiosis, and after *C. albicans* inoculation (Figure 3; Supplementary Tables 1–3). For further analysis, we calculated the Bray-Curtis distances (beta diversity) of fungal species between all samples and subsequently analyzed the similarity (ANOSIM) between different sample groups. In untreated mice, an individual composition of colonizing fungi was observed, with various different species making up the major colonizers (Figure 3A; Supplementary Table 1). This is reflected by the beta diversities, where the comparison of wild type vs. CEACAM1-transgenic mice (Figure 3B) and of cage 1 vs. cage 2 (Figure 3C) gave no significant differences for day −4 (*p* = 0.912 and *p* = 0.334, respectively).

After antibiosis/before *C. albicans* inoculation, all samples from cage 1 revealed *Candida lusitaniae* (also named *Clavispora lusitaniae*) as the major species present, with a mean relative occurrence of 99.8% (Figure 3A; Supplementary Table 2). While *C. lusitaniae* was identified as part of the mycobiota in all samples analyzed in untreated mice at day −4, it only made up for 0.03–2.3% of all OTUs identified (Supplementary Table 1). Samples from cage 2 showed a different picture: the four mice with strongly reduced ITS counts (Figure 1B) displayed a more complex composition of fungal species (Figure 3A). However, the species composition was altered compared to the untreated mice. For the single mouse from cage 2 with high ITS copy numbers (Figure 1B), mouse 2-2, no species could be determined, but *Saccharomycetales* made up for 99.5% of all OTUs. Again, these observations are validated by the analysis of beta diversities: the comparison of wild type vs. CEACAM1-transgenic mice (Figure 3B) showed no differences at day 0 (*p* = 0.318), while cages 1 and 2 (Figure 3C) differed significantly (*p* < 0.001). Not surprisingly, a comparison of both, wild type and transgenic mice, before and after antibiosis (day −4 vs. day 0) showed a significant difference in their beta diversities after antibiosis (*p* < 0.05 for both). Likewise, the alpha diversity in all mice was reduced significantly after antibiosis (Supplementary Figure 1A). All species identified with their relative occurrence for each sample of day 0 are listed in Supplementary Table 2.

After *C. albicans* colonization (day 11), *C. lusitaniae* was reduced to a mean relative occurrence of 15.6% of all species identified in cage 1 and 0.04% in cage 2 (Figure 3A; Supplementary Table 3). *C. albicans* accounted for 81.3% of species in cage 1 and 97.1% in cage 2 (mean relative occurrence). This changed the beta diversities of both, wild type and CEACAM1-transgenic mice, significantly compared to untreated and antibiotics-treated mice (both: *p* < 0.05 compared to day −4 and *p* < 0.01 compared day 0; Figure 3B). *C. albicans* inoculation also further reduced the alpha diversity in all mice significantly (Supplementary Figure 1A). All species identified with their relative occurrence for each sample of day 11 are listed in Supplementary Table 3.

Taken together, while we discovered no differences in the beta diversities between the two genotypes or in untreated mice, there was a strong cage-wise dependency of the mycobiota composition after antibiosis. This effect is known for the bacterial microbiota (McCoy et al., 2017) and underlines the importance of co-housing or alternative adequate measures when studying gut mycobiota.

### Human CEACAM1 Expression Does Not Alter Intestinal *C. albicans* Colonization in a Colonization/Dissemination Model

In order to monitor the gastrointestinal colonization allowed by the antibiotic treatment, fecal fungal CFU contents were analyzed before antibiotic treatment (day −4), before oral *C. albicans* inoculation (day 0), and at days 2, 4, 7, 11, and 14 post-inoculation (Figure 2A; Supplementary Figure 1B). Fungal CFU counts in fecal samples from untreated mice were around 1 × 10^7^ CFU/g feces. Antibiotic treatment resulted in an increase of fungal CFUs of more than one order of magnitude even before oral *C. albicans* inoculation in half of the mice (Figure 2A; Supplementary Figure 1B). The other mice showed a complete or near complete loss of detectable CFUs in their feces. These data verify the findings from the RT-PCR analysis of fungal ITS sequences (Figure 1B). After oral *C. albicans* inoculation, CFU counts are stable around 2 × 10^8^ CFU/g feces in all animals. CFU counts also did not vary between fecal samples from human CEACAM1-transgenic and wild-type mice at any given day. At day 14, also CFU counts of contents from small intestine, cecum, and colon were analyzed (Figures 2B–D). Cecum and colon contents displayed the highest CFU counts similar to numbers found in the fecal samples (~2 × 10^8^ CFU/g), while the contents from small intestines contained 10 times less CFUs (~2 × 10^7^ CFU/g). Again, no differences between the two genotypes were observed for all intestinal contents.

### Cyclophosphamide-Induced Systemic Dissemination of *C. albicans* From the Murine Gut Is Not Affected by Human CEACAM1

**Figure 4:**
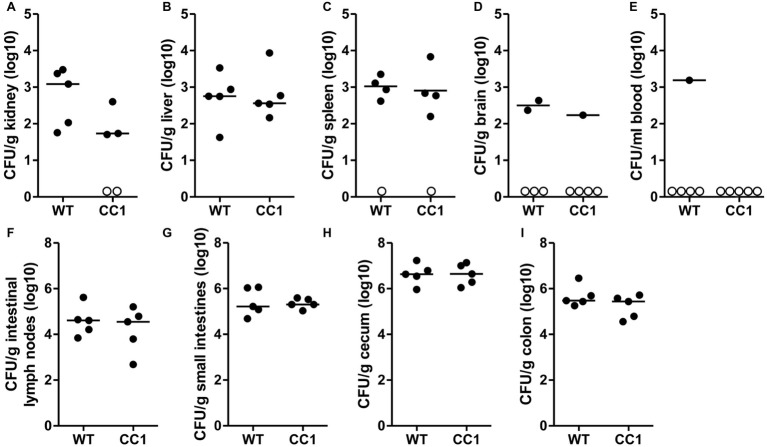
The expression of human CEACAM1 does not influence the cyclophosphamide-induced systemic dissemination of *C. albicans* in a colonization model. Human CEACAM1-transgenic mice (CC1, *N* = 5) and their wild-type littermates (WT, *N* = 5) were treated with antibiotics from day −4, inoculated with 5 × 10^7^ CFU *C. albicans* orally at day 0 and injected with cyclophosphamide at day 11 (see also Table 1). On day 14, mice were sacrificed. Kidneys **(A)**, liver **(B)**, spleen **(C)**, brain **(D)**, and intestinal lymph nodes **(F)** were homogenized directly; small intestines **(G)**, cecum **(H)**, and colon **(I)** were cleared of their contents by rinsing before homogenization (see Figure 2 for the analysis of their contents). Homogenates were analyzed for their CFU content by plating dilutions on YPD/chloramphenicol agar. Blood **(E)** was plated without any homogenization step. Graphs show CFU/g tissue (log10) with median. Numbers of samples without any fungal burden are indicated below the respective groups. Statistical analysis was performed with logarithmized data by unpaired, two-sided Student’s *t* tests (**A**: *p* = 0.064, **B**: *p* = 0.866, **C**: *p* = 0.849, **D**: *p* = 0.322, **E**: no *t*-test possible, **F**: *p* = 0.479, **G**: 0.829, **H**: *p* = 0.972, **I**: *p* = 0.186). All data are from one single experiment.

We next analyzed the *C. albicans* dissemination from the gastrointestinal tract induced by one dose of cyclophosphamide at day 11 post-inoculation. CEACAM1-transgenic mice and their wild-type littermates were sacrificed at day 14, and various organs were analyzed for their CFU counts (Figure 4). Kidneys, livers, and spleens displayed similar fungal burdens of around 1 × 10^3^ CFU/g tissue (Figures 4A–C). However, kidneys from two CEACAM1-transgenic mice and spleens from one CEACAM1-transgenic mouse and one wild-type mouse did not show any fungal burdens. Also, the majority of brain samples (7 of 10) and blood samples (9 of 10) displayed no fungal growth (Figures 4D,E). The intestinal lymph nodes had a higher fungal load of around 1 × 10^5^ CFU/g. The three intestinal samples showed the highest fungal burdens of approximately 5 × 10^5^ CFU/g (small intestine, colon) to 5 × 10^6^ CFU/g (cecum) (Figures 4G–I). No significant differences in the fungal load between the genotypes were detected in all organs tested.

### The Presence of Human CEACAM1 Affects Neither Mouse Survival nor Fungal Burden in a *C. albicans* Blood Stream Infection Model

**Figure 5:**
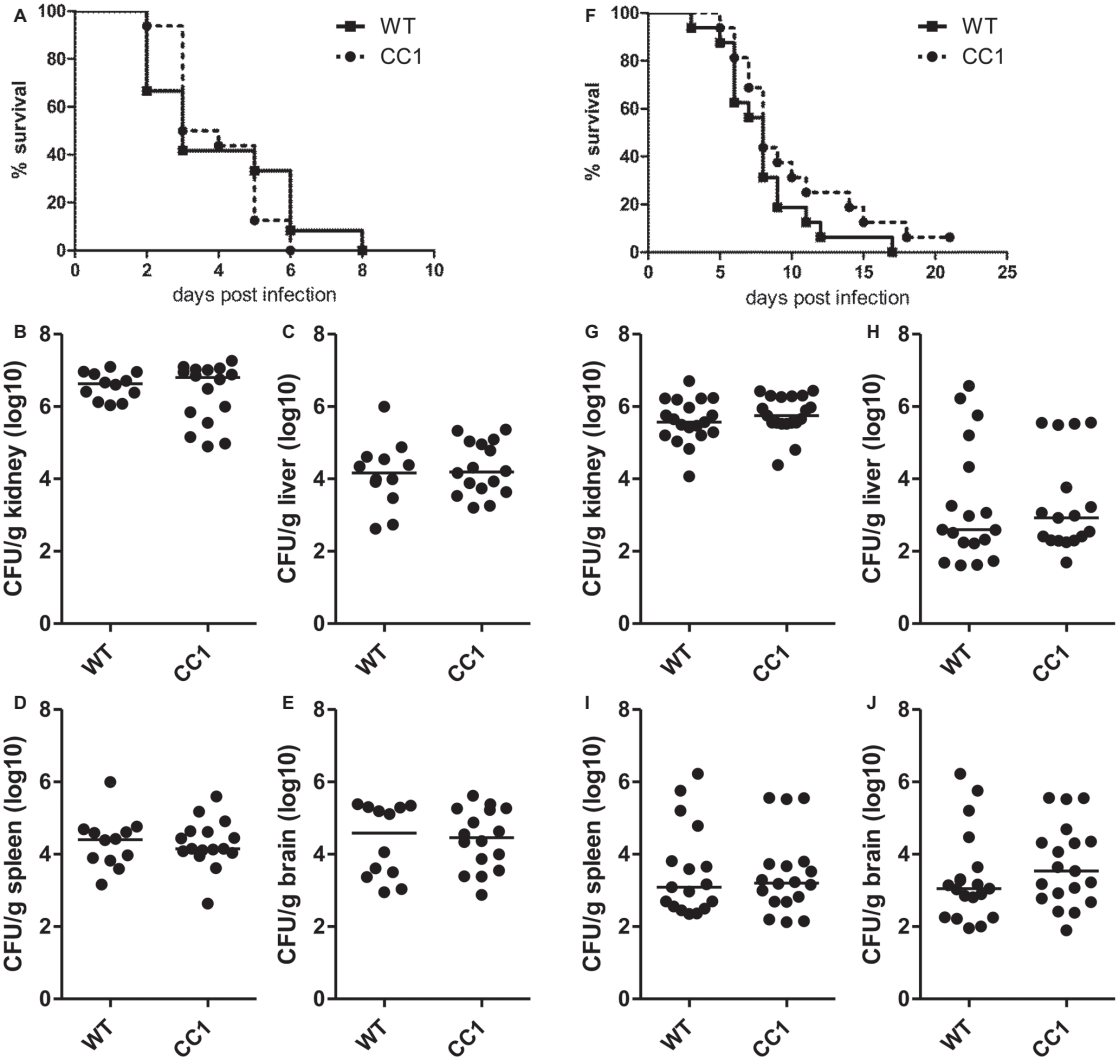
The expression of human CEACAM1 does not affect the susceptibility for systemic candidiasis after tail vein injection. Human CEACAM1-transgenic mice (CC1) and their wild-type littermates (WT) were injected with 2.5 × 10^5^ CFU *C. albicans*/g body weight (**A–E**, corresponding to a total of 5 × 10^6^ CFU for a mouse of 20 g body weight) or 1 × 10^5^ CFU *C. albicans*/g body weight (**F–J**, corresponding to a total of 2 × 10^6^ CFU for a mouse of 20 g body weight) into the tail vein and sacrificed when reaching a humane endpoint. All graphs show combined data from two independent experiments with *N* = 12 (WT/**A–E**) or *N* = 16 (CC1/**A–E**, WT/**F–J**, CC1/**F–J**). **(A,F)** Survival analysis. Note that log-rank tests did not show any significant differences in the survival between the two genotypes for both infection doses (**A**: *p* = 0.660, **F**: *p* = 0.177). Kidneys **(B,G)**, livers **(C,H)**, spleens **(D,I)**, and brains **(E,J)** were removed during necropsy, homogenized, and analyzed for their CFU content by plating dilutions on YPD/chloramphenicol agar. Graphs show CFU/g tissue (log10) with median. Statistical analyses **(B–E,G–J)** were performed with logarithmized data by non-paired, two-sided Student’s *t* tests (**B**: *p* = 0.411, **C**: *p* = 0.626, **D**: *p* = 0.903, **E**: *p* = 0.850, **G**: *p* = 0.344, **H**: *p* = 0.908, **I**: *p* = 0.833, **J**: *p* = 0.372).

Since CEACAM1 is an important immuno-regulatory receptor on various types of immune cells, including myeloid and lymphoid cells, we used a second systemic infection model based on *C. albicans* tail vein injection in order to reduce variances inherent in the model of induced dissemination, i.e., the numbers of CFUs disseminating from the gut into otherwise sterile sites. For survival analysis, mice were infected with 2.5 × 10^5^ CFU *C. albicans*/g body weight (Figures 5A–E, high dose) or 1 × 10^5^ CFU *C. albicans*/g body weight (Figures 5F–J, low dose). Both infection doses resulted in disseminated candidiasis that progressed to lethal disease, and mice were sacrificed when they reached a humane endpoint.

We examined hematological parameters of peripheral blood at the time of death. Untreated mice served as controls. Blood was taken retro-orbital and analyzed in a hemocytometer (Supplementary Figures 2, 3). We identified candidiasis-induced changes in several white blood cell parameters, including an increase in relative neutrophil counts (Supplementary Figure 2G) and a decrease in relative lymphocyte counts (Supplementary Figure 2H). Relative and total monocyte counts showed very high variances within the groups (Supplementary Figures 2D,I). The analysis also revealed platelet-associated differences typical for sepsis like reduced platelet counts and increased platelet volumes (Supplementary Figures 3I,K). However, no differences between the two genotypes were discovered within the treatment groups (WT untreated vs. CC1 untreated; WT endpoint vs. CC1 endpoint) for any of the parameters tested.

As expected, mice infected with the lower *C. albicans* dose survived longer, with a median survival of 8 days for both genotypes, than mice infected with the higher dose, which showed a median survival of 3 days (WT) and 3.5 days (CEACAM1-transgenic), respectively (Figures 5A,F). Log rank tests revealed no significant differences in the survival of human CEACAM1-transgenic and wild-type mice for both infection doses.

At the time of death, kidneys, livers, spleens, and brains were analyzed for their fungal loads (endpoint analysis). As expected for this infection model, kidneys displayed the highest CFU counts (Figures 5B,G). The fungal load of the kidneys differed less than one order of magnitude between the two infection doses, with the higher infection dose resulting in approximately 5 × 10^6^ CFU *C. albicans*/g kidney and the lower infection dose in 1 × 10^6^ CFU *C. albicans*/g kidney. We found no differences in the fungal loads between the two genotypes. Liver, spleen, and brain tissues differed about two orders of magnitude in their fungal loads between the two infection doses; they displayed approximately 1 × 10^4^ to 1 × 10^5^ CFU *C. albicans*/g tissue for the higher infection dose (Figures 5C–E) and around 1 × 10^2^ to 1 × 10^3^ CFU *C. albicans*/g tissue for the lower infection dose (Figures 5H–J). Again, there were no differences in the fungal loads between wild-type mice and human CEACAM1-transgenic mice, respectively.

### *Candida* Infection Results in an Organ-Specific Regulation of CEACAM1 Expression

Since CEACAM1 expression is upregulated on epithelial and myeloid cells during bacterial and fungal infections (Muenzner et al., 2001, 2002; Klaile et al., 2013, 2017; van der Flier et al., 2013), we analyzed expression levels of human CEACAM1 in lysates of kidneys and livers of transgenic mice sacrificed without prior treatment or sacrificed after systemic *C. albicans* infection (1 × 10^5^ CFU *C. albicans*/g body weight; mice from Figures 4E,F).

**Figure 6:**
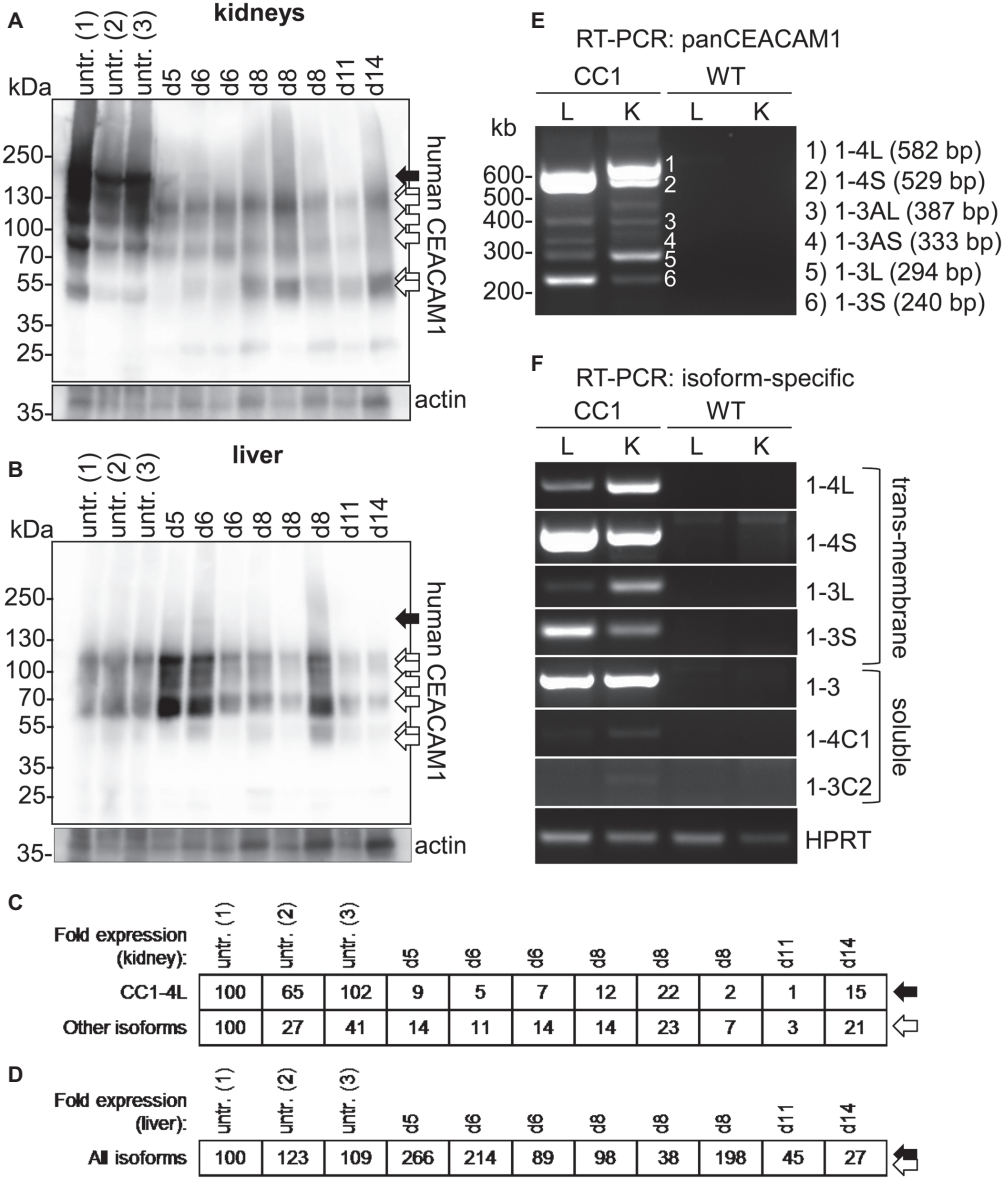
Organ-specific regulation of CEACAM1 expression during systemic *C. albicans* infection. **(A,B)** Human CEACAM1-transgenic mice were either sacrificed untreated (“untr.”) or injected with 1 × 10^5^ CFU *C. albicans*/g body weight (corresponding to a total of 2 × 10^6^ CFU for a mouse of 20 g body weight) into the tail vein and sacrificed when reaching a humane endpoint (mice from Figures 5F–J; “endpoint”). Kidneys **(A)** and livers **(B)** were removed at the time of death and analyzed by Western blot for their human CEACAM1 expression. Actin re-blots served as loading controls. For infected animals, the time of death is given in days above the lanes (d5 to d14). Black arrows indicate the largest isoform, CEACAM1-4L. Open arrows indicate smaller isoforms, which could not be distinguished by Western blot. **(C,D)** Quantification of relative CEACAM1 expression. Please note the lack of cross-reactivity of the anti-human CEACAM1 antibody with mouse CEACAM1 as shown in Supplementary Figure 4. In kidney samples, CEACAM1-4L and all remaining CEACAM1 signals were quantified and adjusted to the respective actin signals **(C)**. In liver samples, total CEACAM1 signals were quantified and adjusted to the respective actin signals **(D)**. Data are representative from one of two experiments shown in Figure 5F. **(E,F)** RT-PCR analysis of human CEACAM1 isoforms expressed in livers (“L”) and kidneys (“K”) of untreated human CEACAM1 transgenic mice (“CC1”). Wild-type mice served as controls (“WT”). **(E)** PanCEACAM1 primers were able to detect all transmembrane isoforms containing exons 4 and 9, resulting in multiple products. Corresponding human CEACAM1 isoforms were assigned to the numbered signals, and the expected product size is given in brackets, respectively. **(F)** Isoform-specific, exon-spanning primers were used to detect seven human CEACAM1 isoforms specifically. Note that the HPRT1-positive controls shown in Panel **(F)** also apply to the panCEACAM1 RT-PCR **(E)**, as all samples were analyzed in parallel. Results are representative for three human CEACAM1 transgenic mice.

In kidneys of uninfected mice, at least seven distinguishable signals for human CEACAM1 were detected (Figure 6A, arrows). The signal with the highest molecular weight was found distinctly above the 130 kDa marker and represents the largest human CEACAM1 isoform, CEACAM1-4L (Figure 6A, black arrow; Gu et al., 2010). The other CEACAM signals (Figure 6A, open arrows) are not easily assigned, since RT-PCR analysis revealed the presence of at least four considerable and two minor additional CEACAM1 isoforms in the kidneys (CEACAM1-4S, CEACAM1-3L, CEACAM1-3S, CEACAM1-3, CEACAM1-3AL, and CEACAM1-3AS; Figures 6E,F). Also, differential glycosylation has a major impact on the apparent molecular weight of the various isoforms. CEACAM1-4L was the major isoform in kidneys of untreated mice and disappeared nearly completely during *C. albicans* infection. Quantification of the CEACAM1-4L signals and signals of the residual CEACAM1 isoforms (Figure 6C) verified a significant loss of all isoforms after *C. albicans* infection (*p* < 0.01 for both).

Similarly, seven different signals were detected for human CEACAM1 in Western blot analysis of liver samples (Figure 6B). As expected (Gu et al., 2010), the CEACAM1-4L isoform was only faintly visible in some samples (Figure 6B, black arrow), and the major signals present were smaller isoforms (Figure 6B, open arrows), mainly consisting of transmembrane isoforms with a short cytoplasmic domain, CEACAM1-4S and CEACAM1-3S, as well as the soluble CEACAM1-3, as shown by RT-PCR analysis (Figures 6E,F). Three of the eight infected transgenic mice showed a higher CEACAM1 expression level in their livers, the other five animals had similar or reduced expression levels compared to the uninfected mice (Figure 6D). In contrast to the kidneys, all detectable CEACAM1 isoforms appeared to be expressed at similar ratios in uninfected and infected animals, and no isoform-specific differences were observed, regardless of the total expression level. Statistical analysis of the CEACAM1 signals revealed no significant differences between total CEACAM1 expression levels in livers from untreated and infected mice (*p* = 0.843).

### The Expression of Human CEACAM1 Does Not Alter the Response of Bone Marrow-Derived Neutrophils to *C. albicans*

**Figure 7:**
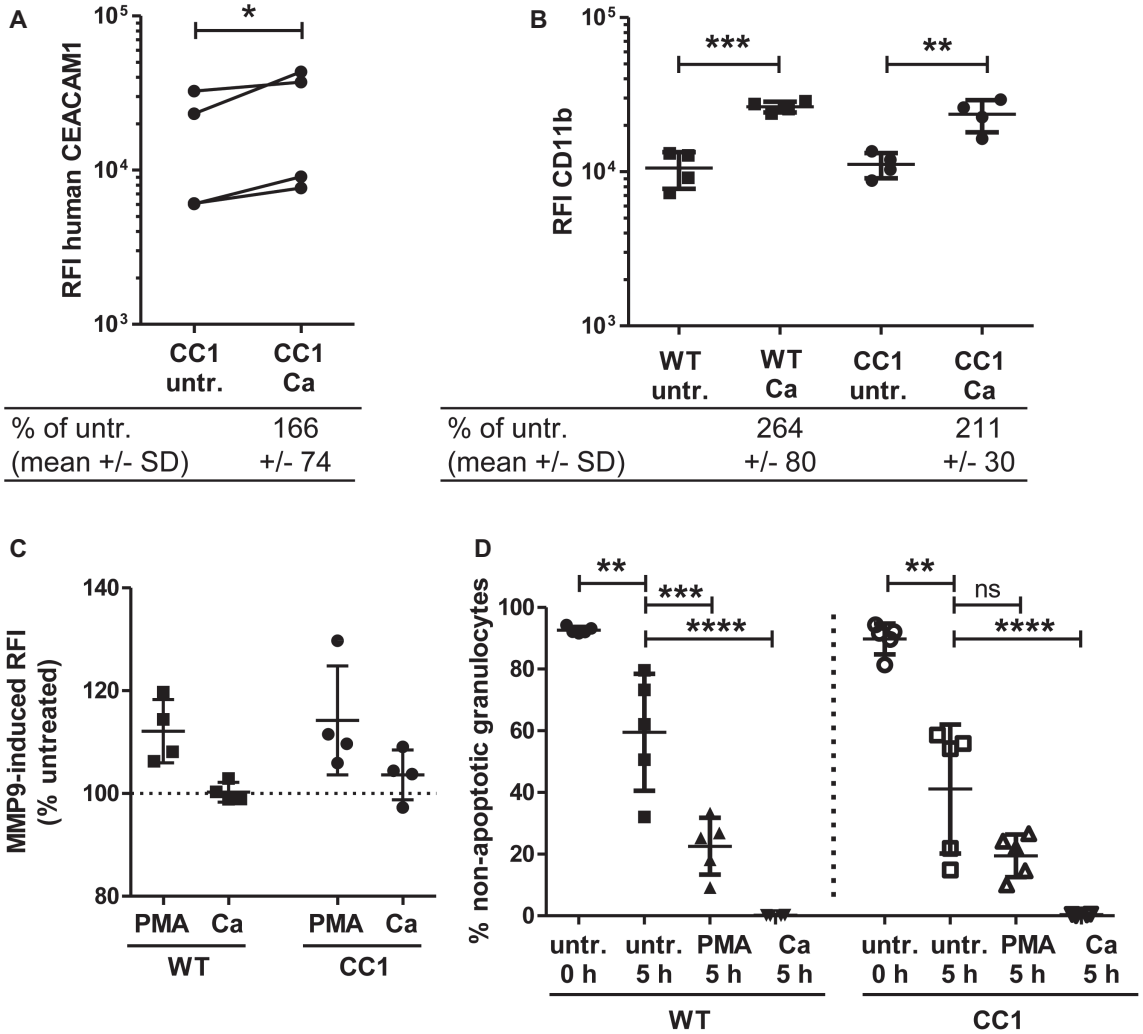
Bone marrow-derived neutrophils (BMNs) from CEACAM1-transgenic mice and WT BMNs have similar cellular properties. BMNs were isolated from femurs of human CEACAM1-transgenic mice (“CC1”) and their wild-type littermates (“WT”). **(A,B)** BMNs were either left untreated (“untr.”) or stimulated with UV-killed *C. albicans* germ tubes (MOI 10; “Ca”) for 60 min and analyzed by flow cytometry for their relative fluorescence intensity (RFI) of human CEACAM1 **(A)** (transgenic BMN only), and of CD11b **(B)**. Below the graphs, the relative expression of the proteins in the Ca-treated samples compared to the untreated samples is given in percent (mean and standard deviation—SD). Statistical analysis was performed by paired, two-sided Student’s *t* test of logarithmized data **(A)** or one-way ANOVA of logarithmized data with Bonferroni post-tests **(B)**. Please note the lack of cross-reactivity of the anti-human CEACAM1 antibody **(A)** with mouse CEACAM1 shown in Supplementary Figure 5. **(C)** BMNs were either left untreated for 0 or 5 h or were treated with 50 ng/ml phorbol myristate acetate (PMA) or with UV-killed *C. albicans* germ tubes (MOI 10; Ca) for 5 h. Spontaneous and PMA-/Ca-induced apoptosis was assessed by Annexin V/PI staining with subsequent flow cytometry analysis; the graph shows non-apoptotic granulocytes (Annexin V- and PI-negative) as % of total granulocytes. **(D)** MMP9 (gelatinase) activity was determined in cell culture supernatants from BMNs that were either left untreated or stimulated with 50 ng/ml PMA or live *C. albicans* yeast cells (MOI 10) for 60 min by measuring the relative fluorescence intensity induced by the degradation of the *EnzCheck DQ-Gelatin*-substrate. Data are given as % activity of untreated cells. Statistical analysis of **(C,D)** was performed by one-way ANOVA with Bonferroni post-tests. *p < 0.05, **p < 0.01, ***p < 0.005, ****p < 0.001, ns = not significant.

Neutrophils play a pivotal role in the host response to *C. albicans* (Lionakis, 2014). CEACAM1 is expressed on their cell surface and regulates different neutrophil functions like adhesion to endothelial cells and apoptosis (Kuroki et al., 1995; Singer et al., 2005; Skubitz and Skubitz, 2011). We therefore studied the reaction of bone marrow-derived neutrophils (BMNs) from human CEACAM1-transgenic mice and their wild-type littermates to *C. albicans*. Since CEACAM1 is also present in secondary/specific granules of human granulocytes and can be degranulated to increase the cell surface expression level upon granulocyte activation (Kuroki et al., 1995; Gu et al., 2010), we first examined the human CEACAM1 expression on transgenic BMNs by flow cytometry. The stimulation of BMNs with *C. albicans* resulted in a small significant increase in human CEACAM1 on the cell surface (Figure 7A).

Next, we analyzed neutrophil functions related to the neutrophil recruitment to infected areas. The integrin CD11b/CD18 (also known as CR3, αMβ2, MO-1, and Mac-1) is important for the neutrophil extravasation by mediating adhesion to endothelial cells and is activated and upregulated during neutrophil activation. Importantly, also CEACAM1 ligation can activate und upregulate CD11b on human neutrophils (Skubitz et al., 2001; Skubitz and Skubitz, 2008). Flow cytometry revealed a *Candida*-induced increase of CD11b on the cell surface of BMNs from both genotypes, but wild-type and CEACAM1-transgenic BMNs had similar CD11b expression levels with and without *C. albicans* stimulation, respectively (Figure 7B). MMP9 (matrix metalloproteinase 9, gelatinase) also plays a role in neutrophil recruitment, facilitating the transmigration through cell layers. In a mouse stroke model, the presence of mouse CEACAM1 inhibits the MMP9-mediated damage to endothelial cells (Ludewig et al., 2013). However, *C. albicans* stimulation did not result in a significant induction of MMP9 activity (Figure 7C). BMNs from both genotypes showed a similar PMA-induced MMP9 release.

Since neutrophils have a short half-life, the regulation of apoptosis can influence neutrophil numbers during infections, affecting pathogen clearance or the resolution of the inflammation (Simon, 2003). We tested for apoptosis behavior in mouse BMNs since CEACAM1 can regulate the viability of rat granulocytes and of human cell lines and primary cells (Singer et al., 2005; Li and Shively, 2013; Zhao et al., 2015). CEACAM1-transgenic and wild-type BMNs displayed no differences in spontaneous apoptosis and in PMA- or *C. albicans*-induced apoptosis (Figure 7D).

**Figure 8:**
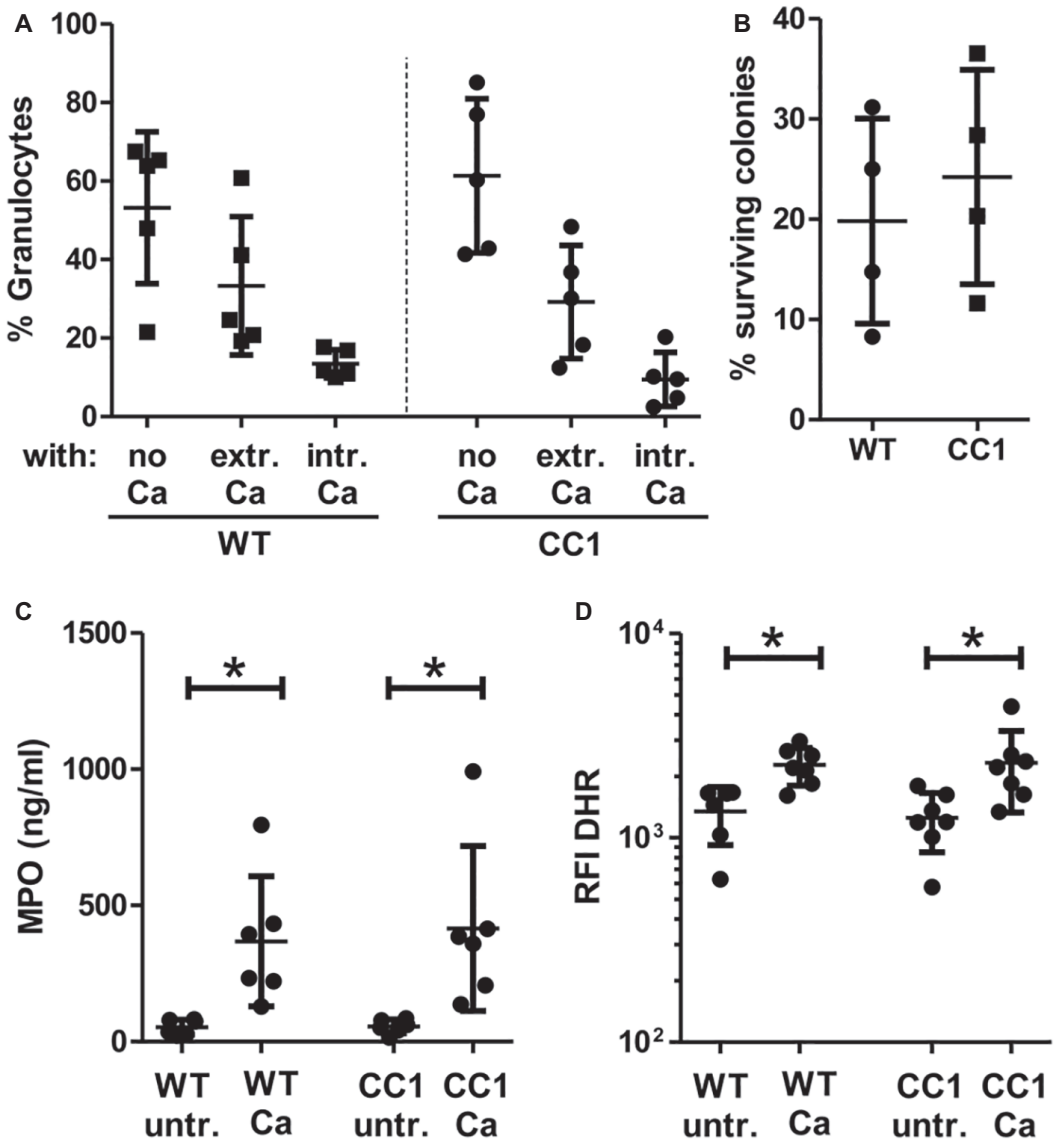
CC1 BMNs do not differ from WT BMNs in their fungicidal reaction to *C. albicans*. BMNs were isolated from femurs of human CEACAM1-transgenic mice (“CC1”) and their wild-type littermates (“WT”). **(A)** The ability of BMNs from both genotypes to bind and phagocytose *C. albicans* was tested by infecting BMNs for 20 min with living, FITC-labeled *C. albicans* yeast cells (MOI 20). Cells were fixed and counterstained by indirect immunofluorescence for mouse CEACAM1 and extracellular *Candida* cells. Micrographs were analyzed for phagocytosis by counting BMNs without contact to *Candida* cells (“no Ca”), BMNs with attached, extracellular *Candida* cells (“extr. Ca”, FITC staining and anti-*Candida* antibody staining), and BMNs with intracellular/phagocytosed *Candida* cells (“intr. Ca”, FITC staining only). Statistical analysis: one-way ANOVA with Bonferroni post-tests. **(B)** Overall candidacidal activity of BMNs from both genotypes was tested by incubating 2.5 × 10^5^ BMNs with 2.5 × 10^4^ *Candida* yeast cells (MOI 0.1) or yeast cells alone for 60 min. *Candida* CFUs were counted after plating different dilutions on YPD agar. Data are shown as % of yeast cells without BMN. Unpaired, two-sided Student’s *t* test (*p* = 0.574). **(C)** Concentrations of released myeloperoxidase (MPO) were determined by ELISA in cell culture supernatants from BMNs that were either left untreated or stimulated with live *C. albicans* yeast cells (MOI 10) for 60 min. Statistical analysis: one-way ANOVA with Bonferroni post-tests. **(D)** In order to measure the induction of reactive oxygen species, BMNs were pre-incubated with dihydrorhodamine 123 (DHR) fluorigenic probe and either left untreated (“untr.”) or stimulated with UV-killed *C. albicans* germ tubes (MOI 10; “Ca”) for 15 min and analyzed by flow cytometry for the relative fluorescence intensity (RFI) of intracellular oxidized DHR. Statistical analysis: one-way ANOVA of logarithmized data with Bonferroni post-tests.

We next analyzed fungicidal responses in the BMNs (Figure 8). Pathogen binding and/or phagocytosis are prerequisites for intracellular and extracellular killing. *C. albicans* binding and phagocytosis were similar in CEACAM1-transgenic and wild-type BMNs (Figure 8A). BMNs of both genotypes also exhibited similar total killing efficiencies (Figure 8B). Myeloperoxidase (MPO) is present in azurophilic (primary) vesicles and is released upon neutrophil activation. It produces cytotoxic hypochlorous acid from hydrogen peroxide and chloride anions (Cl^−^), which kills fungal and other pathogens. While *C. albicans* induced the release of MPO from BMN, no difference between CEACAM1-transgenic and wild-type BMNs was detected (Figure 8C). Similarly, *C. albicans* treatment resulted in a comparable production of fungicidal reactive oxygen species (ROS) in BMNs from both genotypes (Figure 8D). Taken together, human CEACAM1 expression did not alter BMN responses to *C. albicans.*

### Yeast Cells Bind With a Lower Affinity to Human CEACAM1 Than Bacterial Cells

**Figure 9:**
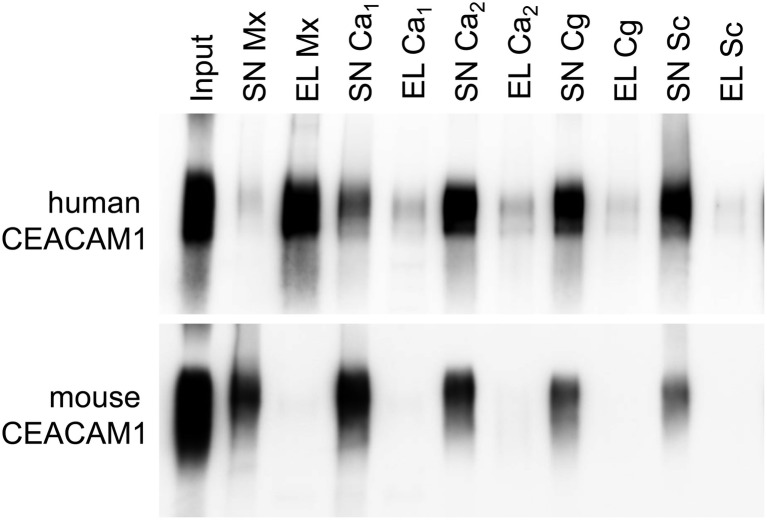
Yeast cells display a lower affinity to human CEACAM1 than the bacterial pathogen *Moraxella catarrhalis*. 2 × 10^8^ live yeast cells (*C. albicans* SC5314, “Ca_1_”; *C. albicans* 28a, “Ca_2_”; *C. glabrata* 2001, “Cg”; *Saccharomyces cerevisiae* BY4741, “Sc”) or *M. catarrhalis* cells (“Mx”) were incubated with 1 μg recombinant human CEACAM1-Fc protein or mouse CEACAM1-His protein (input). Supernatants (SN) were removed, and samples were washed twice. Bound proteins were eluted (EL), and all samples were analyzed by Western blotting for the presence of the respective recombinant protein. One representative experiment of three is shown. Note that for *M. catarrhalis* the majority of the human CEACAM1 protein is found in the eluted fraction, while for all yeast cells, the larger portion of the human CEACAM1 protein remains in the supernatants (upper panel). As expected, no binding was observed for mouse CEACAM1 (lower panel).

For bacterial CEACAM1-binding pathogens like *Helicobacter pylori*, *Moraxella catarrhalis*, *Neisseria meningitidis*, and *Haemophilus influenzae*, the expression of human CEACAM1 in CEACAM-negative epithelial and neutrophil-like cell lines results in an enhanced bacterial binding and/or uptake (Hill and Virji, 2003; Hill et al., 2005; Voges et al., 2010; Sarantis and Gray-Owen, 2012; Javaheri et al., 2016). Also, BMNs from CEACAM1-transgenic animals displayed an increased binding of Opa52 protein (a *Neisseria* CEACAM1-binding protein) expressing *E. coli* (Gu et al., 2010). Since we detected no such increase in the adhesion of *C. albicans* to either human CEACAM1-positive BMNs (Figure 6E) or human CEACAM1-transfected Hela cells (Klaile et al., 2017), we analyzed the relative strength of the interaction between human CEACAM1 and various yeast cells in comparison to *M. catarrhalis* using pulldown assays (Figure 9). Recombinant human CEACAM1-Fc protein bound to a similar degree to all tested yeast cells, including two *C. albicans* strains, a *C. glabrata* strain and an *S. cerevisiae* strain. In contrast to *M. catarrhalis*, where the majority of the human CEACAM1-Fc protein was present in the pulldown eluates, in all yeast samples, the major portion of the human CEACAM1-Fc protein remained in the supernatant. Thus, the recombinant human CEACAM1-Fc protein displayed stronger interactions with *M. catarrhalis* than with yeast cells (Figure 9).

## Discussion

Our recent *in vitro* studies show that the interaction of human CEACAM1 with *C. albicans* is necessary for the CXCL-8 release in an intestinal epithelial cell model (Klaile et al., 2017). We therefore analyzed human CEACAM1-transgenic mice in a *C. albicans* colonization/dissemination model, where the pathogen was first allowed to colonize the gastro-intestinal tract after antibiosis and was induced subsequently to disseminate by the injection of cyclophosphamide. The cyclophosphamide regiment used in the present study resulted in a mild neutropenia and reduced intestinal epithelial barrier functions. In a murine infection model, the transgenic mice demonstrated the important role of human CEACAM1 expression for the intranasal mucosal colonization of the CEACAM1-binding pathogen *Neisseria meningitides* (Johswich et al., 2013). Similar to the *Candida*-induced IL-8 production of the human intestinal mucosal cells, the mouse nasopharyngeal mucosa of human CECAM1 transgenic animals express KC, the mouse homologue of CXCL-8 (interleukin-8), in a human CEACAM1-dependent fashion in response to *Neisseria meningitides* infection (Johswich et al., 2013). Vaginal and uterine colonization models with CEACAM1-binding *N. gonorrhoeae* showed that the expression of human CEACAM1 is fundamental for pathogen adhesion and tissue penetration in mucosae of the female reproductive organs, but the vaginal mucosa did not produce KC (Li et al., 2011; Islam et al., 2018). Contrary to these examples of enhanced colonization of human CEACAM1-transgenic mice by CEACAM-binding bacterial pathogens at three different mucosal surfaces, the presence of human CEACAM1 did not alter the *C. albicans* colonization and dissemination pattern in the murine intestinal colonization model used here.

One possible explanation for this lies within the different niches represented by the gut mucosa and the nasal, vaginal, and uterine mucosae; their discriminative composition; and the disparity in the respective micro- and mycobiota (Krüger et al., 2019). In contrast to other mucosal surfaces, the gut mucosa is protected from direct contact with intestinal microbiota by a thick mucus layer, probably preventing the interaction of CEACAM1 expressed on epithelial cells with pathogens. Also, for bacterial colonization of the other mucosal sites, the strong binding of the pathogen to its CEACAM receptor is of major importance for the bacterial adhesion to the cell surface (Tchoupa et al., 2014). Not only *Neisseria* spp., but also other human pathogens such as *H. pylori*, *M. catarrhalis*, or *Fusobacterium* spp. bind to CEACAM receptors with a very high affinity *via* specific, unrelated proteins (Opa proteins, HopQ, Uspa1, and CbpF, respectively) (Hill and Virji, 2003; Hill et al., 2005; Voges et al., 2010; Sarantis and Gray-Owen, 2012; Javaheri et al., 2016; Koniger et al., 2016; Brewer et al., 2019). We found in the present work and in previous experiments that *C. albicans* and other yeast cells displayed lower affinities for CEACAM1 and that the interaction of *C. albicans* with CEACAM1 – while it induced CEACAM1 phosphorylation and signaling – did not alter the adhesion of *C. albicans* to epithelial cells (Klaile et al., 2017). Therefore, the disparity in the CEACAM1 dependence of the mucosal colonization by bacterial pathogens and *C. albicans* might, in addition to physiological differences between the mucosal tissues, also be related to the different binding affinities of CEACAM1 to the bacterial and fungal surface proteins.

Also, CEACAM1 might adopt a role different from its function(s) during bacterial colonization/infection and rather regulate the intestinal immune homeostasis and the inflammatory response, as we proposed based on our *in vitro* data (Klaile et al., 2017). CEACAM1-mediated effects on the homeostasis as well as on *C. albicans* tissue penetration and dissemination might be difficult to detect, since in contrast to the above mentioned bacterial colonization models, the intestinal mucosa is affected in the *Candida* colonization model by the treatment with cyclophosphamide. This chemotherapeutic facilitates the translocation of *C. albicans* across the intestinal epithelium by damaging the mucosal barrier and immunosuppression. The damage might conceal CEACAM1-specific effects, since the magnitude of a CEACAM1-dependent epithelial response similar to the one we discovered in the enterocytic *in vitro* model, i.e., the increase in CXCL-8 release and the enhanced trans-epithelial electrical resistance (Klaile et al., 2017), is possibly surmounted by the effects caused by cyclophosphamide. Additionally, both antibiotics and cyclophosphamide also affect the microbiota (Alexander et al., 2017), which in turn can impact *C. albicans* colonization (Ranjan and Dongari-Bagtzoglou, 2018; Krüger et al., 2019). The present study also showed no effect of human CEACAM1 expression on the dissemination into various organs upon cyclophosphamide treatment. This might be due to other challenges inherent in this model, i.e., the low total numbers of disseminating *Candida* cells and the high variances in these numbers, which impede the detection of smaller biological effects in general.

However, also after systemic infection *via* the tail vein, which has a lower variance due to the controlled number of *Candida* cells reaching the blood stream, no differences in the systemic response or in the fungal burden of kidneys, liver, spleen, and brain of both genotypes were identified at the time of death. We and many other different groups demonstrated in various *in vitro* and *in vivo* models that CEACAM1 attenuates the host response to pathogens or pathogen-associated patterns (PAMPs) as a (co-) inhibitory receptor: it mitigates responses of neutrophils, T cells, NK cells, and B cells (Pantelic et al., 2005; Nagaishi et al., 2006; Lee et al., 2007; Slevogt et al., 2008; Pan and Shively, 2010; Chen et al., 2012; Lu et al., 2012; Singer et al., 2014; Huang et al., 2015; Jones et al., 2016; Horst et al., 2018b; Gur et al., 2019a,b). So far, no systemic infection model for bacterial pathogens was tested in CEACAM1-humanized mice, but human CEACAM1 was able to replace its mouse orthologue and to regulate immune responses in a viral model for systemic infection (Khairnar et al., 2018). In contrast to this chronic viral infection model, the systemic *C. albicans* infection model is causing an intense hyper-inflammatory response based on the recruitment and activation of inflammatory monocytes and neutrophils to infected organs, and the resulting fatal tissue damage leads to the death of the infected animals (Majer et al., 2012). The attenuating effect of activated CEACAM1 on the host cells might be overcome by this overwhelming pro-inflammatory response and might also be one of the factors resulting in the inability of the study presented here to reproduce an effect seen in cell culture, a far less complex model (Klaile et al., 2017).

The systemic immune response in infected mice is mounted by a number of *Candida* receptors present, including dectin-1, dectin-2, Macrophage Mannose Receptor, DC-SIGN, and Toll-like receptors TLR2, TLR4, TLR7, and TLR9, as well as complement receptors CR3, CR4, and Fc receptors (Duggan et al., 2015). The mouse immune response mediated by these receptors is very effective, and mice are intrinsically *Candida* naïve (Duggan et al., 2015). Healthy mice quickly eliminate *Candida* from the system. Also, their immune cells differ in numbers and functions from their human counterparts (Duggan et al., 2015). Further, it is possible that mouse receptor orthologues of human interaction partners (e.g., mTLR2 and mTLR4) cannot interact with the human CEACAM1 receptor or that the human CEACAM1 transgenic mice simply lack the interaction partners necessary for *C. albicans*-specific CEACAM1-mediated responses. These interaction partners may be other immune receptors regulated by CEACAM1 in human cells (e.g., other CEACAM receptors, TLRs, lectins, or receptors of the integrin family) or adapter molecules and kinases/phosphatases necessary for the signal transduction (Müller et al., 2005; Skubitz and Skubitz, 2008; Slevogt et al., 2008; Muenzner et al., 2010, 2016; Lu et al., 2012; Singer et al., 2014; Schirbel et al., 2019; Zhang et al., 2019). However, the epithelial cells of the transgenic mice contain at least the necessary signaling molecules for a human CEACAM1-mediated alteration of the immune response to the bacterial pathogen *Neisseria meningitidis* (Johswich et al., 2013; Islam et al., 2018). The arguments above must also be applied to the finding that the present study revealed no difference in general granulocyte characteristics between wild type and CEACAM1-expressing bone marrow-derived granulocytes (BMNs), such as apoptosis or CD11b expression cells, as well as for *C. albicans* adhesion, phagocytosis, or killing. In contrast to the present results, human CEACAM1 expressed on mouse BMNs is functional in the recognition of bacterial pathogens and mediates CEACAM1-dependent neutrophil responses (Gu et al., 2010; Sarantis and Gray-Owen, 2012).

Further *in vitro* studies are necessary for the analysis of *Candida*-induced CEACAM1-dependent signaling and the identification of essential proteins. It might be difficult to perform these studies based on mice or murine cells expressing human CEACAM1, since in the murine system especially dectin-1 plays a major role in the response to *C. albicans*, and dectin-1 and CEACAM1 share important signaling molecules in their downstream signaling, e.g., Syk kinase and ZAP-70 kinase (Chen et al., 2008; Drummond and Brown, 2011; Lu et al., 2012; Khairnar et al., 2015). Human granulocytes are a prime candidate to address *Candida*-induced CEACAM1 signaling, since granulocytes are considered the first line of defense against fungal infections (Lionakis, 2014), and various CEACAM1-regulated functions in human granulocytes are published, including apoptosis, adhesion to endothelial cells, and bactericidal/fungicidal responses (Kuroki et al., 1995; Singer et al., 2005; Skubitz and Skubitz, 2011; Lu et al., 2012).

One surprising result of the present study was the isoform-specific regulation of human CEACAM1 in the kidneys of *C. albicans* infected mice. Gu et al. showed that isoforms containing a long cytoplasmic domain (CEACAM1-4L and CEACAM1-3L) are the major isoforms in the kidneys and isoforms containing a short cytoplasmic domain (CEACAM1-4S and CEACAM1-3S) are the major isoforms present in the liver (Gu et al., 2010). Our RT-PCR analysis confirmed these results. However, we could identify the soluble CEACAM1-3 isoform as an additional major isoform in both organs, and especially in the kidneys also minor amounts of the Alu repeat-containing CEACAM1-3AL and CEACAM1-AS were detected on mRNA level.

Since CEACAM1 isoforms comprising the long cytoplasmic domains with their inhibitory motifs are major regulators of immune responses, and the ratio between long and short isoforms affects cellular responses regulated by CEACAM1 (Müller et al., 2009; Dankner et al., 2017; Horst et al., 2018a; Helfrich and Singer, 2019), the loss of the largest (long) isoform in the kidneys during candidiasis might actually influence the immune response toward a stronger pro-inflammatory response with the associated advantages and disadvantages, i.e., enhanced pathogen clearing and increased tissue damage, respectively. As discussed above, these possible regulatory effects of CEACAM1 on the host response are probably overcome by the massive immune response induced by the recognition of *C. albicans* also by other immune receptors.

With regard to the gut mycobiota, we provide new insights into the alterations induced by antibiotic treatment and *C. albicans* colonization, despite the lack of a CEACAM1-specific effect. Six of 10 mice behaved with an increase in fungal numbers to the anti-bacterial antibiotic treatment, as was also found by others (Dollive et al., 2013). This influence of anti-bacterial antibiotics on the increase in the fungal load in general, and of *Candida* species in particular, is also acknowledged in humans (Lewis et al., 2015; Huseyin et al., 2017). To our knowledge, the present study is the first to describe the loss of fungal colonizers together with the eradication of bacteria by antibiosis, as displayed by 4 of 10 animals (all housed in the same cage, cage 2). We can only speculate that the general dysbiosis induced by the antibiotic treatment, which disturbs the close relations of fungal and bacterial commensals (Huseyin et al., 2017), led to this reduction in fungal numbers. The complex cross-kingdom interactions between fungi and bacteria were recently reviewed comprehensively and overstep the scope of this discussion (Krüger et al., 2019). Due to the fact that mice are coprophages, and one mouse in cage 2 (mouse 2-2) displayed a high fungal load of a species belonging to the order of *Saccharomycetales*, it is likely that at a later time point and in the absence of the oral *C. albicans* inoculation used here, all mice within cage 2 would have shown matched high fungal colonization. The general ability of the four mice to harbor high numbers of fungal colonizers is shown by the fungal loads of feces and samples from intestinal contents after oral *C. albicans* administration, which were comparable to the numbers found in the other six mice (Figure 1B).

The finding that antibiosis led to the dominant growth of one species in the gut of all mice within one cage was also observed by others (Dollive et al., 2013). They detected a cage-specific increase in singe species after antibiosis at a comparable time point (2 days of antibiotic treatment, corresponding to day −2 in our experiment). They find *Candida (Clavispora) lusitaniae* to be that major species in two of four cages after antibiosis. The other two cages displayed *Cyberlindnera fabianii* and *Candida tropicalis* as dominant species. Taken together, *Saccharomycetales* may have a general advantage under antibiotic treatment over other fungi found in the gut mycobiota, since our study and the study performed by Dollive et al., despite some differences in the antibiotic regiment, revealed a species belonging to this order as the dominant species in all cages analyzed (Dollive et al., 2013).

Taken together, we found that the presence of human CEACAM1 in transgenic mice was not sufficient to alter the host immune response to *C. albicans* in two different models of systemic candidiasis. This inability to detect CEACAM1-specific differences might be due to deficiencies inherent in the employed mouse models or due to a lack of human interaction partners in the transgenic mice. Still, it remains of major importance to find new targets to modulate host signaling pathways for a better control of *C. albicans* infections (Carpino et al., 2017). Further studies with human and mouse *in vitro* and *in vivo* models will be necessary to shed light on the CEACAM1-yeast interaction with its specific challenges and to identify essential CEACAM1-interacting proteins.

## Data Availability Statement

The raw data (fastq files) supporting the conclusions of this manuscript are deposited on the Sequence Read Archive (SRA) at National Center for Biotechnology Information (NCBI). Data can be accessed at https://www.ncbi.nlm.nih.gov/; accession number: PRJNA551920.

## Ethics Statement

The animal study was reviewed and approved by “Beratende Kommission nach §15 Abs. 1 Tierschutzgesetz” and the responsible Federal State authority Thüringer Landesamt für Verbraucherschutz, Bad Langensalza, Germany (Permit No. 02-019/14).

## Author Contributions

EK and HS conceived the study. EK, MM, SB, SF, TK, AD, CZ-B, TN, SR, and IJ performed the experiments. EK, SB, SF, TK, CZ-B, MS, TN, and HS analyzed the data. EK drafted the manuscript. EK, MM, SB, SF, TK, MS, AD, CZ-B, TN, BS, SR, PZ, IJ, and HS revised and approved the manuscript.

### Conflict of Interest

The authors declare that the research was conducted in the absence of any commercial or financial relationships that could be construed as a potential conflict of interest.

## Footnotes

^1^http://www.earthmicrobiome.org/protocols-and-standards/its/

^2^https://unite.ut.ee/index.php
