# Supplementary material for: Unaltered Fungal Burden and Lethality in Human CEACAM1-Transgenic Mice During Candida albicans Dissemination and Systemic Infection
Source: Front Microbiol. 2019 Nov 26;10:2703. doi: 10.3389/fmicb.2019.02703 (PMC6889641; doi:10.3389/fmicb.2019.02703)
Supplement: Supplementary file 1 [file Data_Sheet_1.docx]

Supplementary Material

# Supplementary Figures and Tables

## Supplementary Figures


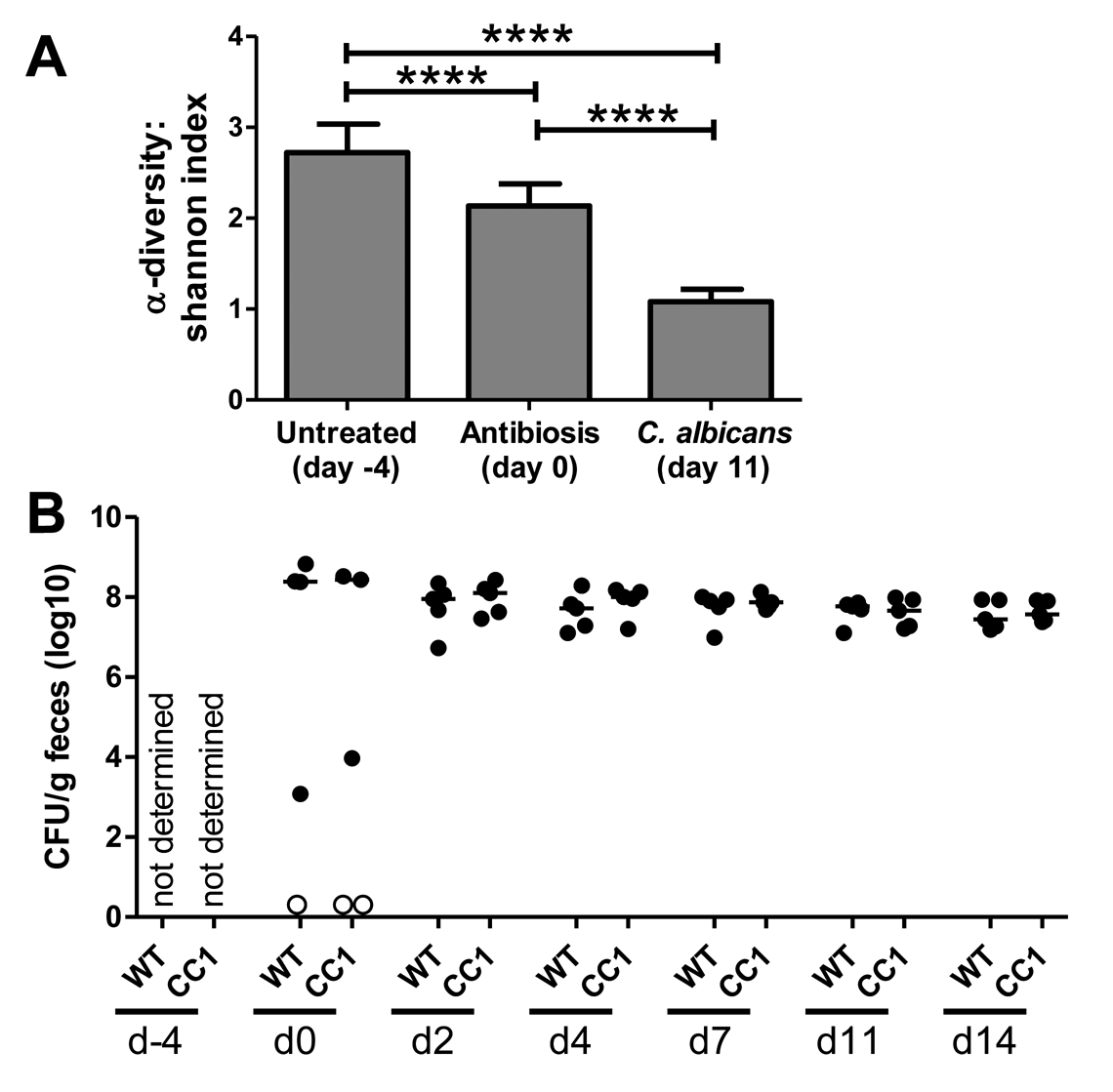


**Supplementary Figure 1.** **The expression of human CEACAM1 does not influence the intestinal colonization by *C. albicans*.** Human CEACAM1-transgenic mice (CC1, N=5) and their wild type littermates (WT, N=5), co-housed in two cages, were treated with antibiotics from day -4, inoculated with 5 × 10^7^ CFU *C. albicans* orally at day 0 and injected with cyclophosphamide at day 11 (see also Table 1). Feces pellets were collected from individual mice. (A) Bar plots of alpha diversity analyses of samples from all mice analyzed in Figure 2. Species diversity was calculated within the different groups (untreated mice /day -4, after antibiosis/day 0, and after oral C. albicans inoculation/day 11) based on the Shannon index. Statistical analysis: one-way ANOVA (****p<0.0001). (B) Feces was collected from individual mice at the days indicated and analyzed for CFU content by plating dilutions on YPD agar containing 80 µg/ml chloramphenicol. Note that similar CFUs were detected on YPD agar, shown in Figure 3A. Open circles represent samples without any CFUs detected by plating on either YPD plate type (detection limit: 17 CFU/g feces; this value was inserted for statistical analysis of samples without detected CFUs). Graphs show CFU/mg feces (log10) with median. One-way ANOVA of logarithmized data with Bonferroni post-test; no differences were detected between the two genotypes at any time. All data are from one single experiment.


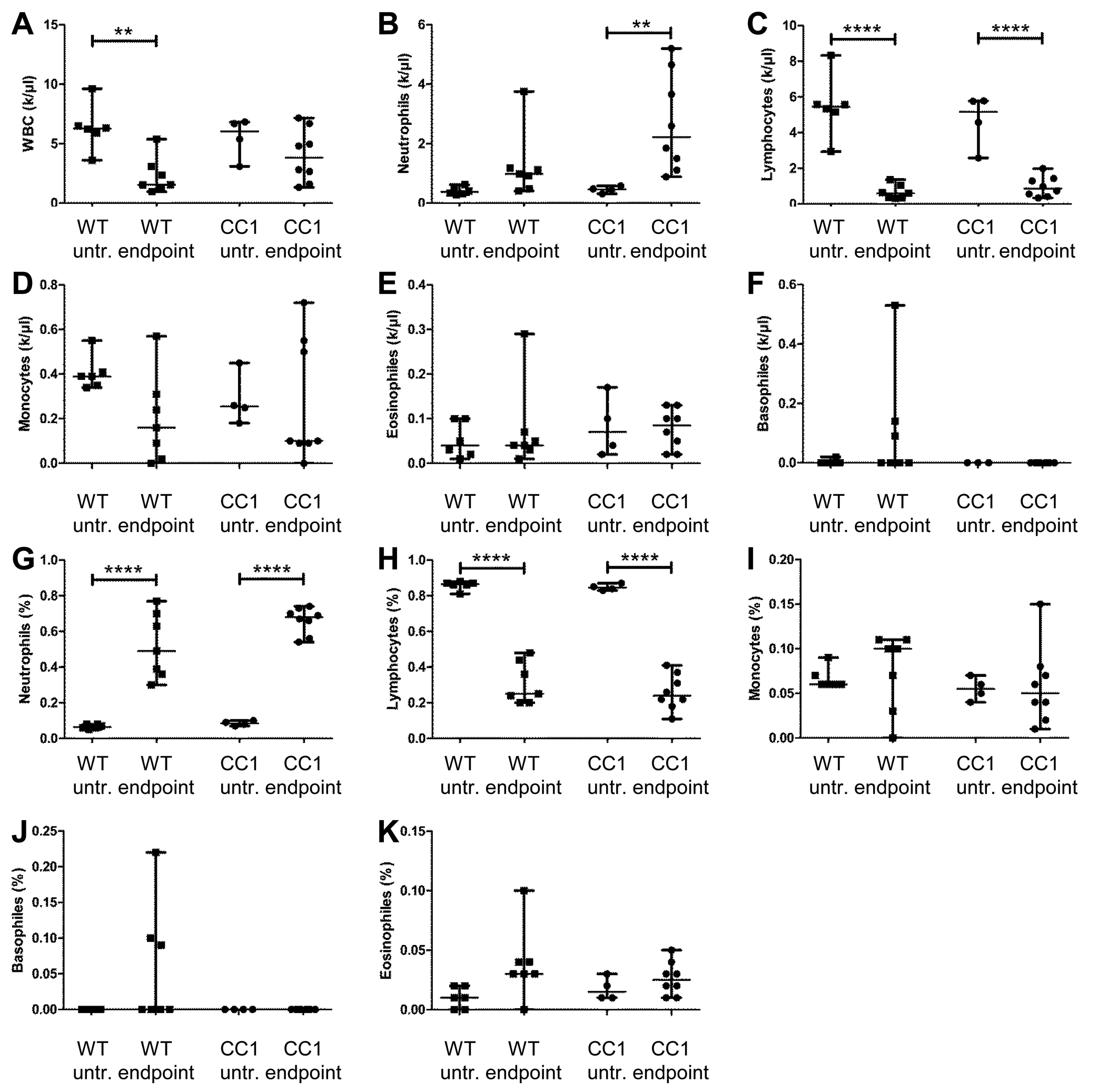


**Supplementary Figure 2: The expression of human CEACAM1 does not influence *C. albicans*-induced changes in hematologic parameters of white blood cells in a systemic candidiasis model.** Human CEACAM1 transgenic mice (“CC1”) and their wild type littermates (“WT”) were either sacrificed untreated (“untr.”) or injected with 1 X 10^5^ CFU *C. albicans*/g body weight into the tail vein and sacrificed when reaching a humane endpoint. Blood was taken retro-orbital at the time of death and analyzed in a hemocytometer. All graphs show data from one experiment out of two shown in Fig. 5 A-E with a total of N=6 (“WT untr.”), N=4 (“CC1 untr.”) N=7 (“WT endpoint”) and N=8 (“CC1 endpoint”). Note that one-way ANOVA analysis with Bonferroni post-test did not reveal any significant differences between WT and CC1 animals from the corresponding treatment groups (WT untreated vs. CC1 untreated; WT endpoint vs. CC1 endpoint); **p<0.01, ****p<0.001. Note that corresponding red blood cell parameters are given in Supplementary Figure 3.


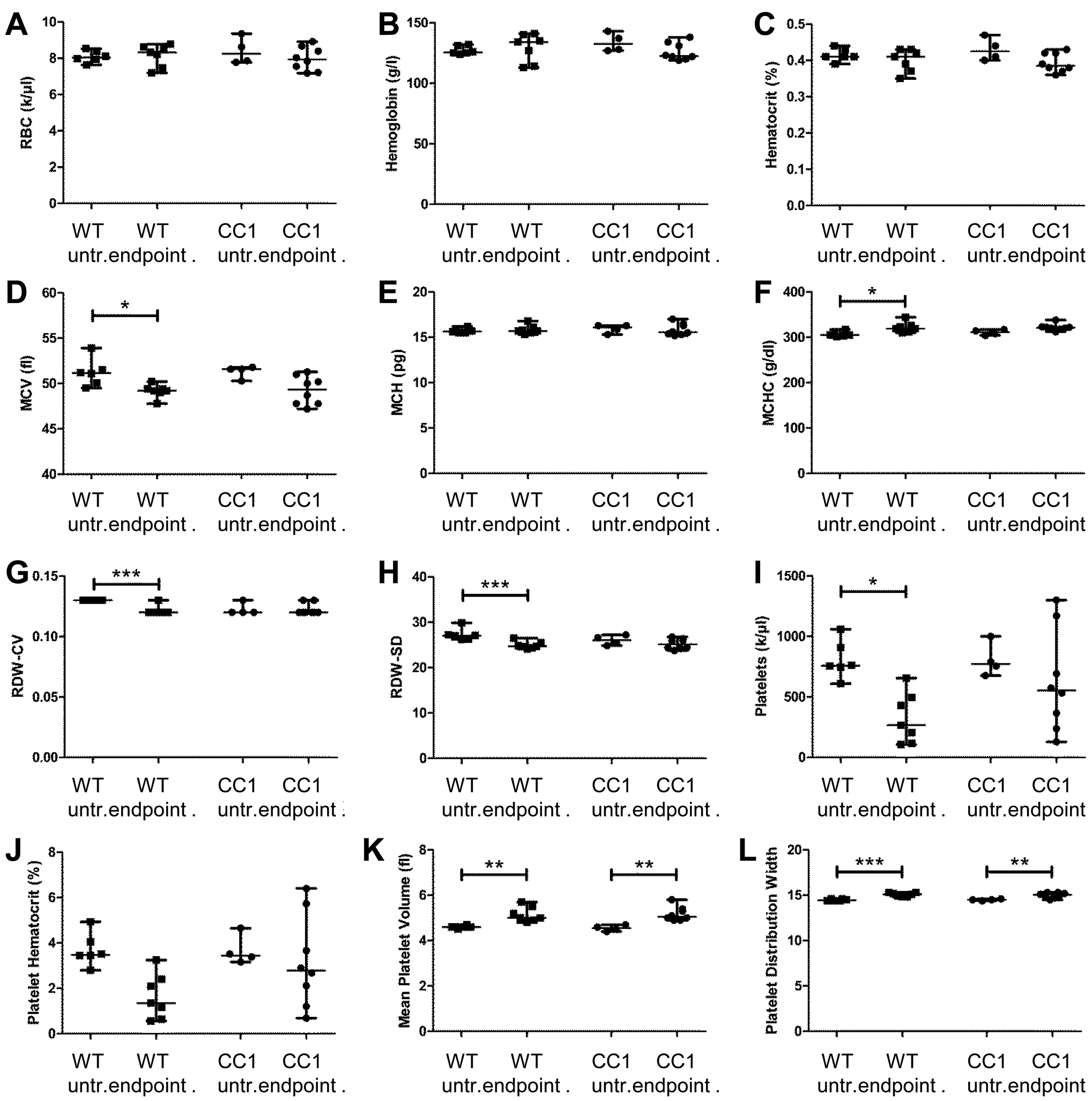


**Supplementary Figure 3: The expression of human CEACAM1 does not influence *C. albicans*-induced changes in hematologic parameters of red blood cells in a systemic candidiasis model.** Human CEACAM1 transgenic mice (“CC1”) and their wild type littermates (“WT”) were either sacrificed untreated (“untr.”) or injected with 1 X 10^5^ CFU *C. albicans*/g body weight into the tail vein and sacrificed when reaching a humane endpoint. Blood was taken retro-orbital at the time of death and analyzed in a hemocytometer. All graphs show data from one experiment out of two shown in Fig. 5 A-E with a total of N=6 (“WT untr.”), N=4 (“CC1 untr.”) N=7 (“WT endpoint”) and N=8 (“CC1 endpoint”). Note that one-way ANOVA analysis with Bonferroni post-test did not reveal any significant differences between WT and CC1 animals from the corresponding treatment groups (WT untreated vs. CC1 untreated; WT endpoint vs. CC1 endpoint); *p<0.05, **p<0.01, ***p<0.005. Note that corresponding white blood cell parameters are given in Supplementary Figure 2.

**
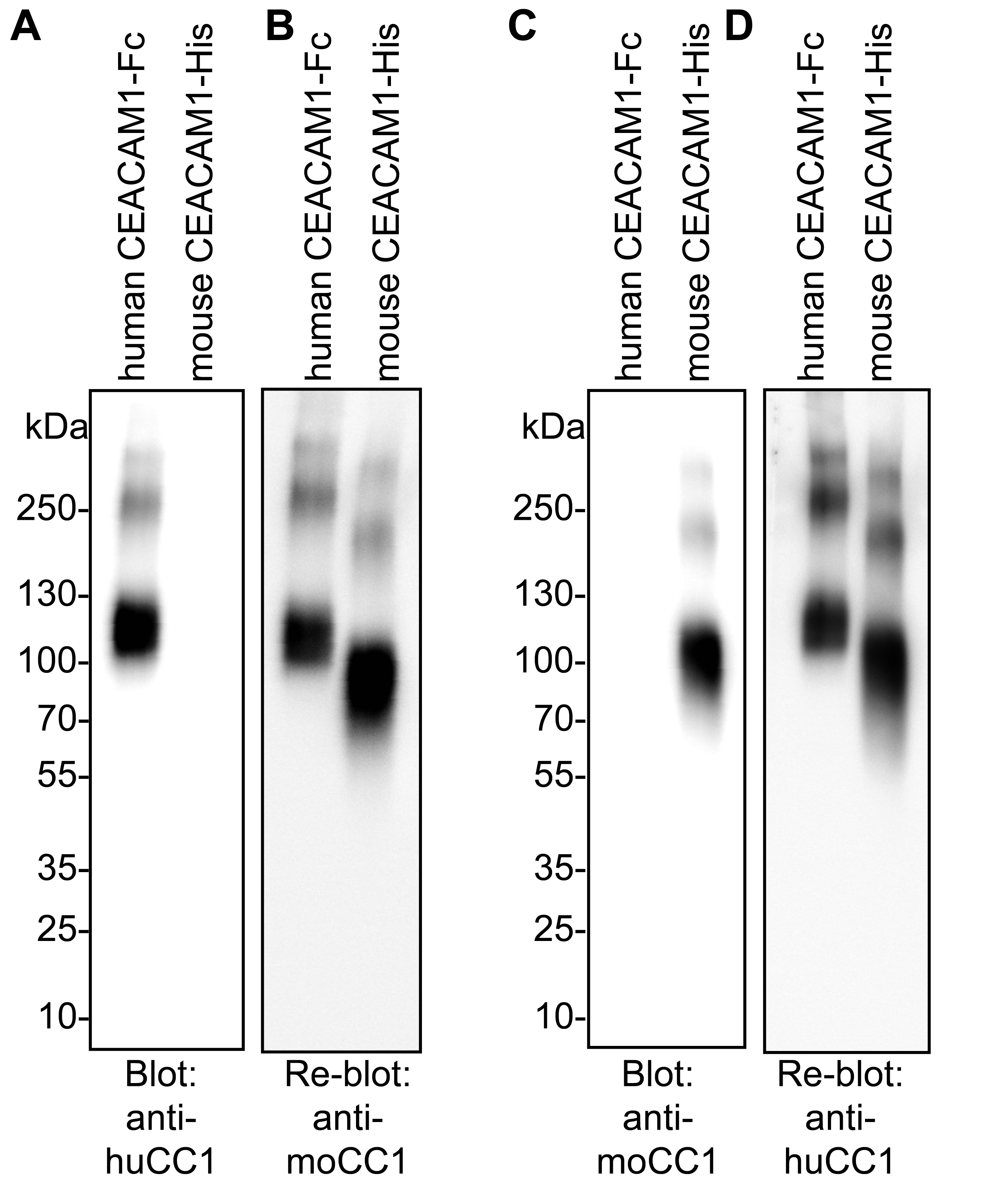
**

**Supplementary Figure 4: Western blot analysis of recombinant human and mouse CEACAM1 proteins for the verification of antibody specificity.** 40 ng of recombinant human CEACAM1-Fc protein (Klaile, et al., 2017, MBio 8; 2) and recombinant mouse CEACAM1-His protein (Hölzel Diagnostika GmbH), both consisting of the respective four IgG-domains and both produced in human cell lines, were blotted and processed in duplicate as described in Section 2.14. The membrane was cut and one half was developed using (A) C5-1X (mouse monoclonal anti human CEACAM1; #101-M181, Reliatech GmbH), and the other half with (C) MSCC1 (mouse monoclonal anti-mouse CEACAM1, Bernhard B. Singer; Essen). The membrane from (A) was re-blotted with MSCC1 (B) and the membrane from (C) was re-blotted with C5-1X (D), resulting in two signals for human and mouse CEACAM1, respectively. Monomeric human CEACAM1-Fc has an apparent molecular weight of about 120 kDa, and monomeric mouse CEACAM1-His of ca. 100 kDa. Signals above 130 kDa are from homo-dimers and -oligomers. Note the lack of cross-reactivity for both antibodies.


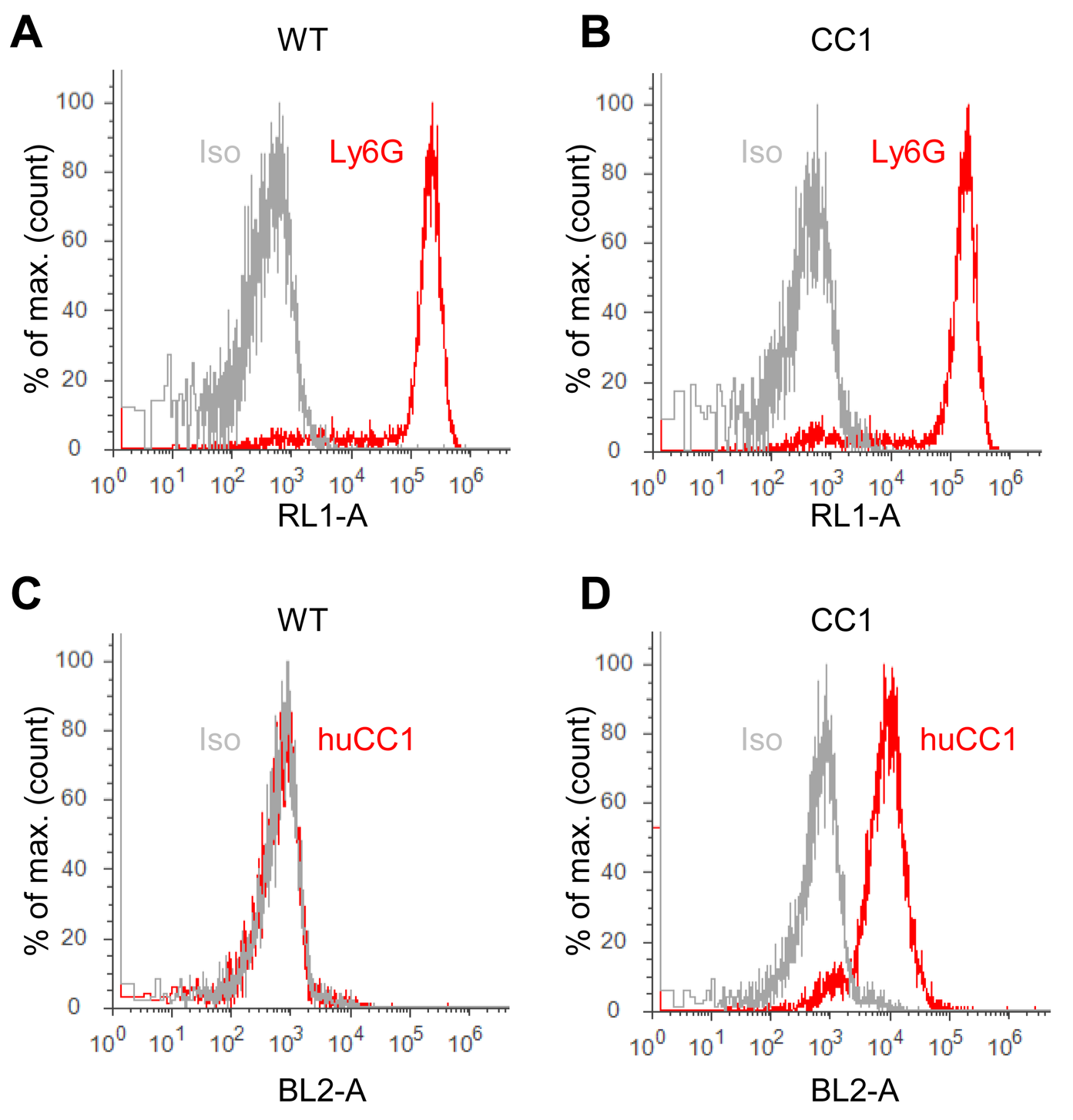


**Supplementary Figure 5: Flow cytometry analysis of bone marrow-derived neutrophils (BMN) from wild type and human CEACAM1-transgenic mice for the verification of antibody specificity.** Bone marrow cells were isolated from CEACAM1 transgenic mice (“CC1”) and their wild type littermates (“WT”) and stained for Ly6G (Anti-Ly-6G-APC, mouse; clone REA526; Milteniy Biotec GmbH) and human CEACAM1 (CD66acde-PE, human, clone REA428; Milteniy Biotec GmbH; also recognizes additional human CEACAM receptors that are not present in the CEACAM1-transgenic mice) or with the respective control antibodies (REA Control PE, clone REA293 and REA control APC, clone REA293, both Milteniy Biotec GmbH) as described in Section 2.18. Panels (A) and (B) show the Ly6G expression (red) over background (isotype, gray) of SSC high/FSC high granulocytes from WT (A) and CC1 cells (B). Panels (C) and (D) show the human CEACAM1 expression (red) over background (isotype, gray) of SSC high/FSC high granulocytes from WT (C) and CC1 cells (D). Note the lack of cross-reactivity of the anti-human CEACAM1 antibody with the WT cells.

## Supplementary Tables

**Supplementary Table 1.** **Fungal species (or genera/families/orders) identified in feces from untreated CEACAM1-transgenic mice (CC1) and their wild type littermates (WT) at day -4.** Human CEACAM1-transgenic mice (CC1, N=5) and their wild type littermates (WT, N=5) were treated with antibiotics from day -4 and inoculated with 5 × 10^7^ CFU *C. albicans* orally at day 0. Feces samples were analyzed for fungal species present by ITS1 sequencing. Values are given as % occurrence of total operational taxonomic units (OTUs) identified above 0.1% in at least one sample; species (or genera/families/orders) were ordered according to their accumulated relative presence in all samples analyzed at all days. Feces samples analyzed were the samples also analyzed in Figures 1, 2 and 3). Please note that all numbers are relative values and do not represent total CFU counts (Figure 3, day -4); the lowest rank identified within the taxonomy is given. Note that for three samples (1-4, 2-2, 2-5) not enough feces was left for this analysis since the samples were necessary to determine the primary effect size, fungal colonization, shown in Figure 3. All data are from one single experiment.

|  | **Cage 1** | | | | | **Cage 2** | | | | |
| --- | --- | --- | --- | --- | --- | --- | --- | --- | --- | --- |
|  | **WT**  (1-1) | **CC1**  (1-2) | **CC1**  (1-3) | **WT**  (1-4) | **WT**  (1-5) | **WT**  (2-1) | **CC1**  (2-2) | **CC1**  (2-3) | **CC1**  (2-4) | **WT**  (2-5) |
| *Candida_albicans* | 0.305 | 0.134 | 0.337 |  | 0.370 | 0.525 |  | 6.081 | 25.615 |  |
| *Candida_lusitaniae* | 0.121 | 0.032 | 0.083 |  | 0.097 | 0.341 |  | 0.058 | 2.315 |  |
| *Mycosphaerella_tassiana* | 39.225 | 28.369 | 22.661 |  | 28.555 | 98.359 |  | 50.870 | 3.907 |  |
| *Saccharomycetales (order)* | 0.076 | 0.020 | 0.068 |  | 0.075 | 0.184 |  | 0.020 | 6.512 |  |
| *Alternaria_betae-kenyensis* | 0.006 | 0.005 | 44.817 |  | 3.581 | 0.026 |  | 6.733 | 8.973 |  |
| *Epicoccum_nigrum* | 0.008 | 54.602 | 6.595 |  | 0.005 | 0.013 |  | 8.873 |  |  |
| *Cladosporium_delicatulum* | 20.819 | 0.042 | 21.919 |  | 11.940 | 0.079 |  | 13.388 |  |  |
| *Sporobolomyces_roseus* | 0.006 | 0.007 | 0.005 |  |  | 0.289 |  | 2.914 | 33.864 |  |
| *Fomitopsis_pinicola* | 32.205 |  | 0.002 |  |  | 0.013 |  |  |  |  |
| *Saccharomyces_cerevisiae* | 0.003 |  | 0.007 |  | 0.005 | 0.013 |  |  | 0.289 |  |
| *Wallemia_tropicalis* | 0.003 | 12.547 | 0.005 |  | 9.538 | 0.039 |  | 0.005 | 8.249 |  |
| *Candida_tropicalis* | 0.006 |  | 0.002 |  | 23.804 |  |  |  |  |  |
| *Aspergillus_intermedius* | 0.076 | 0.005 | 0.015 |  | 0.011 | 0.013 |  | 0.006 |  |  |
| *Cryptococcus_pseudolongus* | 0.006 |  | 0.015 |  | 10.208 | 0.039 |  | 2.964 | 5.499 |  |
| *Kluyveromyces_marxianus* |  |  | 0.005 |  |  |  |  | 1.159 | 0.724 |  |
| *Filobasidium_stepposum* | 0.003 |  | 3.119 |  | 11.790 |  |  | 0.001 |  |  |
| *Neoascochyta_graminicola* |  |  | 0.005 |  | 0.005 |  |  |  |  |  |
| *Hannaella_luteola* |  |  |  |  |  |  |  |  |  |  |
| *Alternaria_infectoria* | 0.003 | 0.591 | 0.002 |  |  | 0.053 |  | 0.766 | 4.052 |  |
| *Cyberlindnera_rhodanensis* | 7.128 |  |  |  |  |  |  |  |  |  |
| *Epicoccum_plurivorum* |  | 3.646 |  |  | 0.011 |  |  | 0.001 |  |  |
| *Aspergillus_flavus* |  |  |  |  |  |  |  |  |  |  |
| *Articulospora (genus)* |  |  | 0.002 |  |  |  |  | 3.090 |  |  |
| *Neoascochyta_desmazieri* |  |  |  |  |  |  |  | 3.067 |  |  |
| *Cladosporium_halotolerans* | 0.003 |  | 0.335 |  | 0.005 | 0.013 |  | 0.004 |  |  |

**Supplementary Table 2.** **Fungal species (or genera/families/orders) identified in feces from CEACAM1-transgenic mice (CC1) and their wild type littermates (WT) after antibiosis at day 0.** Human CEACAM1-transgenic mice (CC1, N=5) and their wild type littermates (WT, N=5) were treated with antibiotics from day -4 and inoculated with 5 × 10^7^ CFU *C. albicans* orally at day 0. Feces samples were analyzed for fungal species present by ITS1 sequencing. Values are given as % occurrence of total operational taxonomic units (OTUs) identified above 0.1% in at least one sample; species (or genera/families/orders) were ordered according to their accumulated relative presence in all samples analyzed at all days. Feces samples analyzed were the samples also analyzed in Figures 1, 2 and 3). Please note that all numbers are relative values and do not represent total CFU counts (Figure 3, day -4); the lowest rank identified within the taxonomy is given. All data are from one single experiment. # represents values below 0.001.

|  | **Cage 1** | | | | | **Cage 2** | | | | |
| --- | --- | --- | --- | --- | --- | --- | --- | --- | --- | --- |
|  | **WT**  (1-1) | **CC1**  (1-2) | **CC1**  (1-3) | **WT**  (1-4) | **WT**  (1-5) | **WT**  (2-1) | **CC1**  (2-2) | **CC1**  (2-3) | **CC1**  (2-4) | **WT**  (2-5) |
| *Candida_albicans* | 0.070 | 0.102 | 0.048 | 0.077 | 0.121 | 7.452 | 0.134 | 16.129 | 13.171 | 23.770 |
| *Candida_lusitaniae* | 99.820 | 99.577 | 98.654 | 99.126 | 98.502 | 45.052 | 0.070 | 8.546 | 4.379 | 18.406 |
| *Mycosphaerella_tassiana* | 0.012 | 0.054 | 0.024 | 0.008 | 0.033 | 8.674 | 0.091 | 0.015 | 9.265 | 11.092 |
| *Saccharomycetales (order)* | 0.011 | 0.017 | 0.815 | 0.034 | 0.070 | 0.063 | 99.543 | 2.507 | 13.284 | 12.111 |
| *Alternaria_betae-kenyensis* | 0.022 | 0.060 | 0.016 | 0.024 | 0.139 | 11.682 | 0.077 | 11.314 | 35.991 | 0.033 |
| *Epicoccum_nigrum* | 0.004 | 0.003 | 0.002 | 0.046 | # | 0.010 | 0.002 | 17.921 | 0.006 | 0.360 |
| *Cladosporium_delicatulum* | 0.011 | 0.037 | 0.009 | 0.030 | 0.018 | 5.007 | 0.012 | 7.104 | 2.688 | 0.023 |
| *Sporobolomyces_roseus* | # | # |  | 0.006 |  | 0.023 |  | 1.562 |  | 22.516 |
| *Fomitopsis_pinicola* |  |  |  |  |  |  |  |  |  |  |
| *Saccharomyces_cerevisiae* | 0.003 | 0.081 | 0.004 | 0.030 | 0.011 | 13.202 | 0.036 | 8.580 | 4.224 | 2.343 |
| *Wallemia_tropicalis* | # |  | 0.002 | 0.001 | 0.002 | 0.005 | 0.003 | 0.005 | 0.001 | 0.012 |
| *Candida_tropicalis* |  |  |  |  |  |  | # |  |  |  |
| *Aspergillus_intermedius* | 0.004 | 0.005 | 0.003 | 0.012 | 0.056 | 0.002 | 0.002 | 5.337 | 14.979 | 0.680 |
| *Cryptococcus_pseudolongus* | # | # | 0.009 |  |  | 0.002 | 0.024 |  | 0.001 |  |
| *Kluyveromyces_marxianus* | 0.040 | 0.017 | 0.410 | 0.553 | 1.027 | 0.002 | 0.002 |  |  |  |
| *Filobasidium_stepposum* |  |  |  | # | 0.002 |  |  | 0.002 |  |  |
| *Neoascochyta_graminicola* |  | 0.025 |  | 0.003 | 0.012 | 5.050 | # | 2.898 | 2.004 | 3.759 |
| *Hannaella_luteola* |  |  | # | 0.019 |  | 0.007 |  | 8.585 |  | 3.209 |
| *Alternaria_infectoria* |  | 0.020 | # | 0.006 |  | 3.762 |  | 2.019 |  | 0.005 |
| *Cyberlindnera_rhodanensis* |  |  |  |  |  |  |  |  |  |  |
| *Epicoccum_plurivorum* |  |  | 0.002 | 0.009 | 0.002 |  | 0.002 | 2.101 |  |  |
| *Aspergillus_flavus* |  |  |  | 0.011 | 0.002 |  |  | 5.373 |  |  |
| *Articulospora (genus)* |  |  |  |  |  |  |  |  |  |  |
| *Neoascochyta_desmazieri* |  |  |  | # |  | 0.002 |  |  |  |  |
| *Cladosporium_halotolerans* | # | # | # | 0.001 | 0.002 | 0.005 | # |  | 0.006 | 1.681 |

**Supplementary Table 3.** **Fungal species (or genera/families/orders) identified in feces from CEACAM1-transgenic mice (CC1) and their wild type littermates (WT) after oral *C. albicans* inoculation at day 11 (continuous antibiosis).** Human CEACAM1-transgenic mice (CC1, N=5) and their wild type littermates (WT, N=5) were treated with antibiotics from day -4 and inoculated with 5 × 10^7^ CFU *C. albicans* orally at day 0. Feces samples were analyzed for fungal species present by ITS1 sequencing. Values are given as % occurrence of total operational taxonomic units (OTUs) identified above 0.1% in at least one sample; species (or genera/families/orders) were ordered according to their accumulated relative presence in all samples analyzed at all days. Feces samples analyzed were the samples also analyzed in Figures 1, 2 and 3). Please note that all numbers are relative values and do not represent total CFU counts (Figure 3, day -4); the lowest rank identified within the taxonomy is given. All data are from one single experiment. # represents values below 0.001.

|  | **Cage 1** | | | | | **Cage 2** | | | | |
| --- | --- | --- | --- | --- | --- | --- | --- | --- | --- | --- |
|  | **WT**  (1-1) | **CC1**  (1-2) | **CC1**  (1-3) | **WT**  (1-4) | **WT**  (1-5) | **WT**  (2-1) | **CC1**  (2-2) | **CC1**  (2-3) | **CC1**  (2-4) | **WT**  (2-5) |
| *Candida_albicans* | 75.685 | 86.043 | 83.042 | 79.521 | 81.930 | 98.577 | 96.672 | 96.482 | 97.860 | 95.717 |
| *Candida_lusitaniae* | 23.198 | 11.564 | 14.233 | 11.201 | 17.560 | 0.044 | 0.030 | 0.015 | 0.028 | 0.060 |
| *Mycosphaerella_tassiana* |  |  |  |  |  |  |  |  |  |  |
| *Saccharomycetales (order)* | 0.007 | 0.009 | 0.031 | 0.061 | 0.014 | 1.348 | 3.249 | 3.347 | 2.080 | 3.881 |
| *Alternaria_betae-kenyensis* |  |  |  |  |  |  |  |  |  |  |
| *Epicoccum_nigrum* |  |  |  |  |  |  |  |  |  |  |
| *Cladosporium_delicatulum* |  |  |  |  |  |  |  |  |  |  |
| *Sporobolomyces_roseus* |  |  |  |  |  |  |  |  |  |  |
| *Fomitopsis_pinicola* |  |  |  |  |  |  |  |  |  |  |
| *Saccharomyces_cerevisiae* | 1.085 | 0.122 | 0.016 | 0.173 | 0.118 | 0.026 | 0.040 | 0.150 | 0.016 | 0.013 |
| *Wallemia_tropicalis* |  |  |  |  |  |  |  |  |  |  |
| *Candida_tropicalis* |  |  |  |  |  |  |  | # |  |  |
| *Aspergillus_intermedius* |  | 0.001 |  |  |  |  |  |  |  |  |
| *Cryptococcus_pseudolongus* | 0.022 |  |  |  |  |  |  |  |  |  |
| *Kluyveromyces_marxianus* | 0.004 | 2.260 | 2.679 | 9.044 | 0.377 | 0.004 | 0.010 | 0.005 | 0.015 | 0.328 |
| *Filobasidium_stepposum* |  |  |  |  |  |  |  |  |  |  |
| *Neoascochyta_graminicola* |  |  |  |  |  |  |  |  |  |  |
| *Hannaella_luteola* |  |  |  |  |  |  |  |  |  |  |
| *Alternaria_infectoria* |  |  |  |  |  |  |  |  |  |  |
| *Cyberlindnera_rhodanensis* |  |  |  |  |  |  |  |  |  |  |
| *Epicoccum_plurivorum* |  |  |  |  |  |  |  |  |  |  |
| *Aspergillus_flavus* |  |  |  |  |  |  |  |  |  |  |
| *Articulospora (genus)* |  |  |  |  |  |  |  |  |  |  |
| *Neoascochyta_desmazieri* |  |  |  |  |  |  |  |  |  |  |
| *Cladosporium_halotolerans* |  |  |  |  |  |  |  |  |  |  |
